# Residential proximity to transport facilities as urban determinants of individual-level per- and poly-fluoroalkyl substance (PFAS) exposures: Analysis of two longitudinal cohorts in Singapore

**DOI:** 10.1186/s12940-025-01257-5

**Published:** 2026-01-09

**Authors:** Lucas Shen, Subhashni Raj, Youssef Oulhote, Damaskini Valvi, Sharon Ng, See Ling Loy, Shiao-yng Chan, Tarik Benmarhnia, Jonathan Y. Huang

**Affiliations:** 1 Institute for Human Development and Potential, Agency for Science, Technology and Research (A*STAR), Singapore, Singapore; 2 University of Hawai’i at Mānoa, Honolulu, USA; 3 Icahn School of Medicine at Mount Sinai, New York, USA; 4 KK Women’s and Children’s Hospital, Singapore, Singapore; 5 Department of Obstetrics & Gynaecology, Yong Loo Lin School of Medicine, National University of Singapore, Singapore, Singapore; 6 University of California San Diego, San Diego, USA; 7 Department of Public Health Sciences, University of Hawaiʻi at Mānoa, Honolulu, United States; 8 Centre for Quantitative Medicine, Duke-NUS Medical School, Singapore, Singapore

**Keywords:** PFAS, Built environment, Airborne, Bus terminal, Environmental epidemiology, Urban, Exposures in pregnancy

## Abstract

**Background:**

Policy-relevant spatial determinants of human exposure to Perfluoroalkyl Substances (PFAS), a broad class of persistent environmental contaminants affecting pregnancy and child development, remain poorly understood because of the diversity of exposure sources. This is especially true for modern, dense urban settings, which contain less well-studied built environment-related sources, including transportation-related ground and airborne contamination.

**Methods:**

We link high-resolution spatiotemporal urban land use data to longitudinal residential histories to assess determinants of individual-level blood plasma PFAS exposures in two geographically- and demographically- diverse cohorts of pregnant women in urban Singapore (n = 784 in 2009–2011; n = 384 in 2015–2017). Longitudinal repeated measures allow us to rule out socio-behavioral factors (e.g., residential segregation) as alternative explanations. Actual land use occupancies were ground-truthed through automated extraction of Google Street View data.

**Findings:**

Adjusting for known predictors and within-neighborhood unobserved spatial heterogeneity, a standard deviation (SD) increase ($\sim$10,000m$^2$) in transport facility exposure was linked to 0.11 (1.78 ng/mL), 0.16, 0.11 SD increases in residents’ perfluorobutane sulfonic acid (PFBS), perfluorobutanoic acid (PFBA), and perfluorononanoic acid (PFNA) concentrations, respectively, in the 2009 cohort. Dose-response analyses suggested that associations strengthened when transport facilities exceeded 10,000 m$^2$, with residents living near $\ge$12,000 m$^2$ exhibiting 7.3 ng/mL higher plasma PFBS (p = 0.04), consistent with footprints from large bus depots rather than smaller petrol kiosks. Associations with different PFAS congeners were replicated in the 2015 cohort. No other land use type showed similarly consistent findings.

**Interpretations:**

Transport facilities are prevalent near residences in urban settings and may be potential sources of PFAS emissions from automotive-related lubricants, parts, and materials. Our findings that exposure was robustly associated with individual-level concentration, over and above behavioral and other factors, highlight the importance of monitoring these and other urban sources of exposure.

**Supplementary Information:**

The online version contains supplementary material available at 10.1186/s12940-025-01257-5.

## Introduction

Per- and poly-fluoroalkyl substances (PFAS) include thousands of chemically stable and highly persistent chemicals used in consumer products and industrial applications with potential adverse health effects [1, 2] including lower fertility [3]. Prenatal exposure to PFAS is adversely linked to thyroid function [4]. Newborns with higher in-utero exposure to PFAS have lower birth weight [5–7]. In children, PFAS is linked to airway and respiratory infections [8, 9], emotional-behavioral problems [10], and cognitive performance [11].

Most studies on environmental determinants of PFAS exposure have focused on a few sources, including freshwater reservoirs or wastewater, industrial sites [12–22], and known high-risk land uses such as airports, firefighting training centers, and military bases [23]. Fewer studies focus on specific non-occupational determinants within dense urban residential or commercial areas [18], even though significantly higher PFAS have been found in urban areas [15]. In such urban environments, transport facilities (e.g., bus terminals, transit stations, and petrol stations) are of potential concern because of the potential PFAS burden coupled with their closer proximity to residential and commercial areas where city dwellers spend the majority of their time. PFAS are used extensively in automotive applications (e.g., car bodies, windshield wiper fluid, engine and steering systems, engine oil coolers, cylinder head coatings and hoses, electronics, fuel lines, steel hydraulic brake tubes, interior components, brake pad additives, as well as petroleum-based products and their containers) [24]. In fact, the number of use categories in transport-related automotive products is more than any other known industrial branch (e.g., chemical or semiconductor industries) [24].

Recent work has shown the importance of better accounting for airborne sources of PFAS exposure [25–28], of which automotive uses may be a major source. There is a growing appreciation for the fact that these PFAS are present in the atmosphere, including gases, aerosols, and particulate matter [29–31], which may be exacerbated in urban areas [32–39]. PFAS have been detected in air samples near high-profile sites with PFAS contamination [35, 40–43]. However, it is understudied whether transport facilities are substantial contributors in the urban environment. Furthermore, given wide differences in exposure-related behaviors in adults, particularly pregnant women, direct ascertainment of individual-level exposures is preferred.

We study individual-level plasma PFAS measurements in pregnant and preconception women participants of two population- and geographically-representative longitudinal cohorts in Singapore, a dense, diverse, high-income city in Southeast Asia with relatively high background PFAS levels [19, 44, 45]. Importantly, while regulatory buffers ensure that known exposure sources, such as heavy industrial zones, are distant from residential areas (e.g., Figure A1) shows general industrial land use on the outskirts of residential areas), transport facilities lack such buffers and can be located close to residences. Leveraging high-resolution spatiotemporal administrative data on urban land use, we examine whether individual-level PFAS concentrations are related to proximity to transport facilities and other land use. Using future residence as negative controls, we rule out socio-behavioral factors (e.g., high-risk individuals choosing to live in certain neighborhoods) as explanations for associations with exposure levels.

## Methods

### Study population

This study includes pregnant women from the “Growing Up in Singapore Towards healthy Outcomes” (GUSTO) longitudinal birth and family-follow-up cohort [46]. In total, 1450 pregnant women aged 19–47 were recruited into the study at 7–11 gestational weeks of pregnancy between June 2009 and October 2010. The participants are ethnically and geographically diverse and represent all major residential areas of Singapore (Appendix A.A). We locate participants by linking residential postcodes to planning areas and subzones (more granular) based on administrative borders delineated in the Singapore Urban Redevelopment Authority 2008 Master Plan. Based on the 2010 census, the density of GUSTO participants was representative of 20–49 year-old women at both subzone ($\hat{r} \;=\; 0.89$, $p \;<\; .001$, $n \;=\; 166$ subzones) and planning area ($\hat{r} \;=\; .92$, $p \;<\; .001$, $n \;=\; 33$ planning areas; see Appendix A.A) levels, suggesting geographically representativeness. 

### PFAS measurements

PFAS were analyzed from maternal plasma samples collected at delivery using ultra-performance liquid chromatography-tandem mass spectrometry (UPLC-MS/MS). In total, 11 PFAS were quantified in 784 participants [47]. Eight had concentrations above limits of detection (LOD) and limits of quantification (LOQ) for at least 95% of participants (Table A2). We subsequently included these eight PFAS measurements in our analyses: perfluorobutane sulfonic acid (PFBS) [LOD = 0.078; LOQ = 5 ng/ml], perfluorobutanoic acid (PFBA) [0.41; 0.5 ng/ml], perfluorononanoic acid (PFNA) [0.016; 0.1 ng/ml], perfluorooctanoic acid (PFOA) [0.009; 0.1 ng/ml], perfluorooctane sulfonic acid (PFOS) [0.027; 0.182 ng/ml], perfluorohexane sulfonic acid (PFHxS) [0.024; 0.1 ng/ml], perfluoroundecanoic acid (PFUnDA) [0.011–0.1 ng/ml], and perfluorodecanoic acid (PFDA) [0.01; 0.1 ng/ml]. Measures for these PFAS falling below the LOQ, but above the LOD, were retained as reported by the instrument. Measures falling below the LOD were imputed at LOD/$\sqrt{2}$ [3, 48].

### Residential exposure to transport facilities

**Fig. 1:**
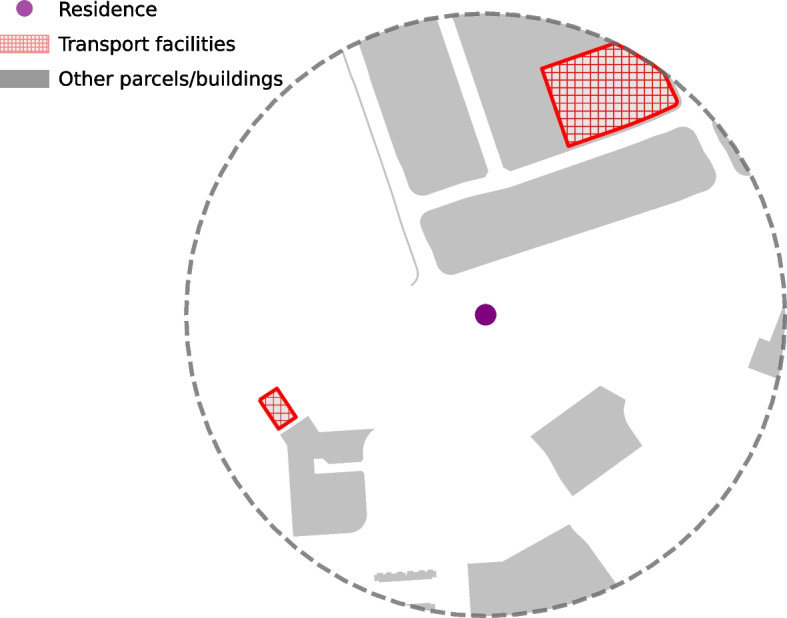
Quantifying residential exposure to transport facilities. Residential exposure to transport facilities is computed based on the area of transport facilities parcels intersecting with the concentric circle (500-meter radius) within 500m ($\sim$1,640 feet) of the residence. The footprint of the transport facility outside the circle is not counted

Built environment shapefiles were obtained from data.gov.sg based on the 2008, 2014, and 2019 Urban Redevelopment Authority Master Plans. The primary exposure was computed as the total area or footprint (measured in square meters) of parcels zoned for transport facilities (bus terminals, petrol stations) within a 500-meter ($\sim$1,640 feet) radius around the centroid of the participant’s residence postcode at the time of birth (Fig. 1). Residential postcodes in Singapore typically include only one or several buildings within a multi-family apartment complex. For secondary and sensitivity analyses, we also computed this measure using alternative buffer radii (e.g., 1000m) and using postcodes reported at different years (other than the year of birth).

### Auditing land parcels for transport facilities

**Fig. 2:**
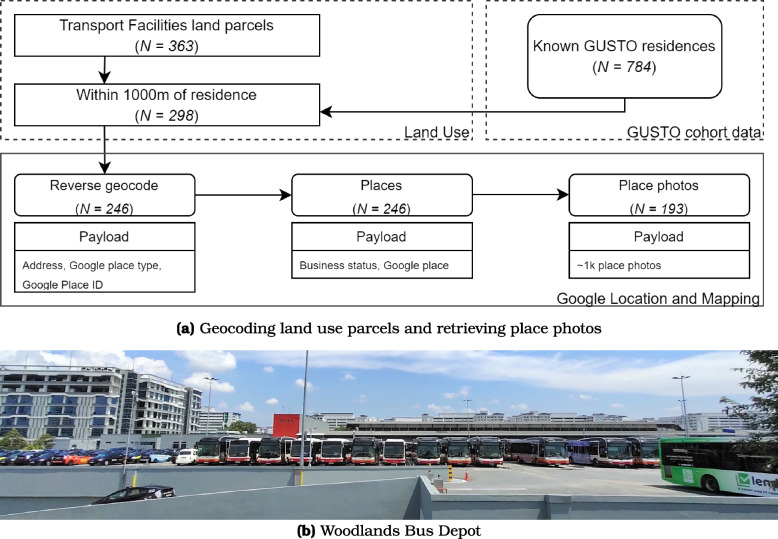
Auditing land parcels for transport facility land use. Panel **A** shows a schematic representation of geo-referenced parcels used to obtain street-level photos of the location. Panel **B** shows an example of a large transport depot (with residential buildings in the backdrop)

To validate planned versus actual land use and to improve interpretation and construct validity, we curated street-level public-contributed images using Google Maps (Fig. 2; Appendix B). Using the geo-referenced land parcels, we query the Place API using the PlaceID before finally querying the Photos API to obtain the street images of the relevant transport facility parcels. We then manually scanned all images. From this approach, most transport facilities appear to be petrol kiosks/gas stations and larger public transit terminals (e.g., bus depot in Fig. 2). Other forms of transportation land use such as subway stations, airports, or parking structures, were not included in this land use type.

### Individual-level (maternal) covariates

The GUSTO cohort collected self-reported sociodemographics and medical history from study-staff-administered interviews with standardized questionnaires at recruitment and mid-pregnancy. Such factors may influence both neighborhood choice and other determinants of PFAS (e.g., diet), including: age at delivery, parity [3, 49–51], place of birth [45, 49, 52], ethnicity (Chinese, Indian, Malay) [23, 45, 49, 53, 54], highest education level [45, 53, 55], occupation [23, 49, 56–58], housing type, marital status, maternal and household monthly income (Table A1) [59, 60].

### Descriptives and summary statistics

We quantified land use parcels over time, including their count, land area, and share of land cover. To better characterize land use around residences, we restricted computations to known residential buildings (around 10k postal codes and close to 80% of the resident population). For PFAS measurements, we computed summary statistics, including percent detected, medians, and interquartile ranges.

### Statistical analyses

**Fig. 3:**
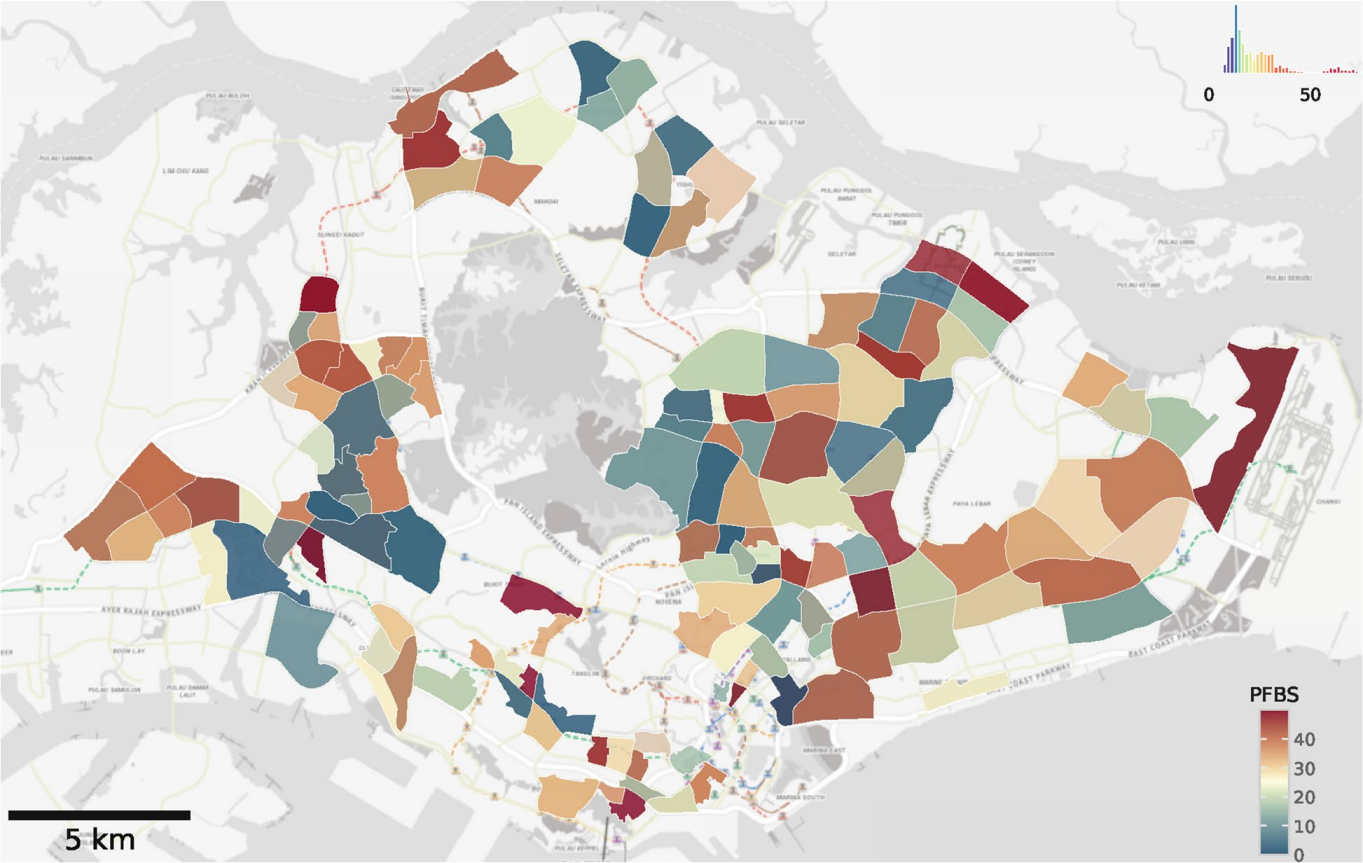
PFBS (GUSTO cohort) averaged across neighborhoods (n = 147). Legend indicates underlying distribution

The relationship of interest is the association between the total transport facility surface area within 500 meters of residence and the average plasma PFAS concentration. We modeled this relationship for each of the eight PFAS by multivariable regression models that adjust for both individual-level maternal characteristics and unobserved, time-invariant spatial determinants of PFAS represented by the administrative area using the form:

1 $$\begin{aligned} \text {PFAS}_{ic} &= \beta (\text {Exposure to transport facilities})_{ic} \\&+ \boldsymbol{\gamma }^{T} \boldsymbol{X}_{i} + \delta _c (\text {administrative area})_c + \varepsilon _{ic}, \end{aligned}$$

where PFAS measure varies by individual *i*, $\boldsymbol{X}_{i}$ as a vector of individual-level maternal characteristics listed above, and the administrative area *c*. The primary assumption is that any systematic variation in individual PFAS concentrations that is not due to exposure to transport facilities is captured by measured individual-level characteristics or the other underlying characteristics of the neighborhood (including the net effect of other land use types). Model 1 adjusts for maternal covariates and the planning areas. Model 2 adjusts for subzones (neighborhoods; Fig. 3), as a more granular level of administrative area. Adjusting for neighborhoods imposes that all participants in the same neighborhood have a fixed underlying average PFAS concentration, and accounts for these differences between neighborhoods, such as drinking water sources or proximity to coasts and water catchments [19, 23, 61]. For all models, standard errors are clustered at the planning area level, recognizing that planning-area delineations strongly influence urban planning and may lead to spatially correlated exposures. This approach provides robust standard errors that account for potential within-area correlation.

We focus on PFBS, an unregulated shorter 4-carbon PFAS replacement previously shown to be disproportionately high in Singapore [1, 45, 62–65]. PFBS is also known to have high concentrations in the region and can remobilize into the air [39]. Analyses of environmental samples from the same city, likely reflecting industrial shifts to the shorter-chain PFAS, show that PFBS (followed by PFBA) has the highest concentrations [19] and is frequently detected across matrices at levels up to 100 times higher than in other cities [44, 66]. Regional studies likewise identify PFBS as the dominant PFSA in air samples [41], and data from nine other Asian cities indicate a relative affinity for PFBS to aerosolize into fine particulate matter [44, 67]. That said, all quantified PFAS are analyzed and reported (Appendix F).

#### Dose-response modeling

In a secondary model, we attempt to quantify a dose-response or non-linearity in transport facility exposure effects. This model was motivated by three observations arising from the main analyses that point to bus terminals and not petrol stations as the primary contributors to observed associations: 1) the two main types of transport facilities (Auditing land parcels for transport facilities section), gas stations and transport depots, have large differences in footprint; ii) our main analyses involving a standard deviation increase in exposure area were already larger than the footprint of a typical gas station (Table A2), and iii) specific influential points were attributable to a single large transport depot (Fig. 6). In response, we conducted ad hoc dose-response modeling varying the definition of exposure to transport facility parcel to be whether neighborhood transport facilities exceeded a specific threshold of land area. We fit models with indicators for exposure to transport facilities varying the threshold *t* (0 to 20,000m$^2$, in steps of 2,000m$^2$):

2 $$\begin{aligned} \text {PFAS}_{ic}& = \beta ^{(t)} {1}\{\text {Exposure to transport facilities> t}\}_{ic}\\& +\gamma X_{i} + \delta _c (\text {administrative area})_c + \varepsilon _{ic}. \end{aligned}$$

Other than substituting the actual land area with a threshold, covariates and models were otherwise similar to Eq. (1) and the main analyses.

### Sensitivity analyses

#### Model specifications

We perform a suite of sensitivity analyses to test our assumptions and rule out potential sources of bias in our non-experimental study. First, we fitted an additional Model 3, adding adjustments for income to Model 2 at the cost of a substantially smaller sample size (Appendix A.B) [59, 60]. Second, we performed jackknife tests to detect influential points at the individual, subzone/neighborhood, and planning area levels (Appendix A.B). Third, we systematically rule out other geographical variations in transport facilities as simply capturing other land use types by explicitly accounting for each other type in turn (Appendix C; Residential exposure to transport facilities seciton). Fourth, we considered neighborhood-level covariates, including other industrial and transport-related land use (Appendix C), neighborhood affluence from housing resale microtransactions at the level of 0.1 km$^2$ [68] and urban pedestrian density from anonymized mobile phone traces smoothed to 0.1 km$^2$ spatial bins (Appendix A.B) [68–70]. Finally, we investigate several alternative means of quantifying exposure to transit facilities, including by commuting time (Appendix D).

#### Negative control exposure

The primary threat to the association between the built environment and individual-level PFAS concentration is if unmeasured sociobehavioral characteristics related to the neighborhood of residence confound the relationship. For example, if individuals who have relatively lower dietary PFAS exposure choose or are otherwise able to live in areas with fewer bus depots, estimates would be biased. To rule out this possibility, we conduct “negative control exposure” analyses to detect the presence of such unmeasured confounding [71]. Specifically, if proximity to transport facilities acutely affects individual PFAS levels, the association should only be seen between PFAS measures and transport facility exposures closely preceding it in time. However, suppose we also observe similar associations between PFAS and transport facilities near residences ten years in the future amongst participants who have moved. In such a case, we may not be able to rule out that unmeasured individual-level characteristics or preferences could explain observed associations. Thus, future transport facility exposure serves as a negative control exposure to help detect whether such bias is present (Appendix E).

#### Replication study

Finally, we use a secondary cohort, the Singapore Preconception Study of Long-Term Maternal and Child Outcomes (S-PRESTO; $N \;=\; 384$) [3, 72], to replicate our findings. S-PRESTO draws from the same geographic area but with younger, preconception women (aged 18–45 at recruitment) recruited at a later period (2015–2017). Strengths of this replication cohort include that (i) it shared a similar recruitment process targeting the same population (Appendix F.B) [3, 72], (ii) it analyzed plasma PFAS measurements from blood samples collected in preconception, and (iii) we were able to construct the exposure measures in an identical fashion using the 2014 master plan.

## Results

### Descriptive statistics

Median (IQR) of plasma PFBS observed in the study was 18.2 ng/mL (13.28–28.97; Table A2). PFBS showed marked geographic patterning (Fig. 3), including in the Eastern-most region adjacent to the commercial international airport [15, 21, 23, 73]. Another area with high PFBS measurements is in the North-West Region (see residential buffer from Fig. 1). Other spatial distributions of PFBS and proximity to transport facilities are given in Appendix A.A (Figures A2 to A7 map GUSTO residences and transport facility footprints across all regions).

### Main analytic findings

**Fig. 4:**
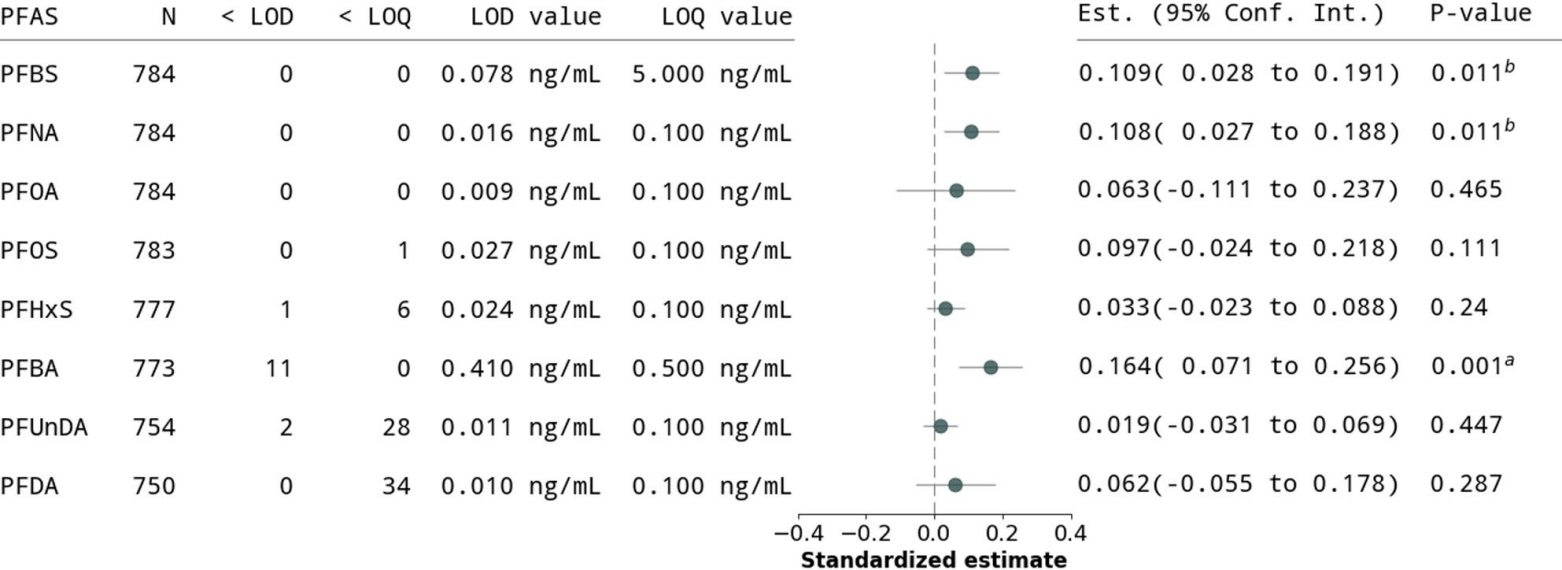
Figure reports all estimates from modeling PFAS measurement (from cord blood) as dependent on exposure to transport facilities. Horizontal axis of the plot reports the $\hat{\beta }$ coefficients from estimating Model 2: $\text {substance}_{ic}^{\text {(scaled)}} = \beta (\text {Exposure to transport facilities})_{ic}^{\text {(scaled)}} +\gamma X_{i} + \delta _c (\text {neighborhood})_c +\varepsilon _{ic},$ where $\text {substance}_{ic}^{\text {(scaled)}}$ is the PFAS substance (indicated in the first column). The PFAS substances and the exposure to transport facilities variable are scaled to have a standard deviation of one. The specification is otherwise identical to that in Eq. (1). Measurements below LOD and LOQ values (indicated in the second and third columns) are first imputed by the LOD/LOQ values (indicated in the fifth and sixth columns) divided by $\sqrt{2}$. Gray horizontal lines are the 95% confidence intervals constructed from standard errors clustered at planning areas. Significance levels: $^c$ 0.1 $^b$ 0.05 $^a$ 0.01 [74]

Comparing individuals within broad planning areas (Model 1), we observed statistical evidence of association between transport facilities and PFAS. This pattern persisted when comparing within neighborhoods (Model 2), with 0.16 ng/mL higher PFBS per 1000m$^2$ increase in transport facilities ($\hat{\beta}$ = 0.150, 95% CI: 0.038–0.261, SE = 0.055, p = .011, Table A4). Further adjusting for income at the cost of a smaller sample size (Model 3), produced similar results ($\hat{\beta}$ = 0.142, 95% CI: 0.028–0.257, SE = 0.056, p = .016). In standardized coefficients, a SD increase in transport facilities ($\sim$11,900m$^2$) is associated with a 0.11 SD (1.78 ng/mL) increase in PFBS. We find similar associations with PFNA (0.11 SD, 0.06 ng/mL) and PFBA (0.16 SD, 0.70 ng/mL; Fig. 4).

**Fig. 5:**
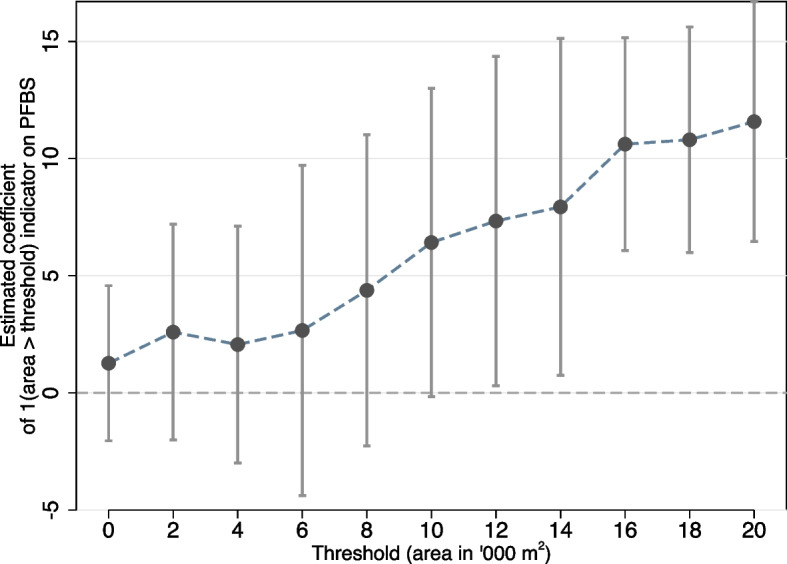
The estimated effect of exposure to an area of transport facilities based on cutoffs from estimating Eq. 2. Each point represents the point estimate from a model that regresses PFBS on an indicator for whether the area of transport facilities within a 500m radius exceeds the area indicated on the horizontal axis. For instance, the first point is the estimate of the binary indicator for area of transport facilities > 0 m$^2$, the second point for area > 2,000 m$^2$, etc. After 20,000 m$^2$, fewer than 50 households would be binned into the exposed group. Capped vertical lines are 95% confidence intervals from clustered standard errors. Please see Appendix F.A for all other PFAS

**Fig. 6:**
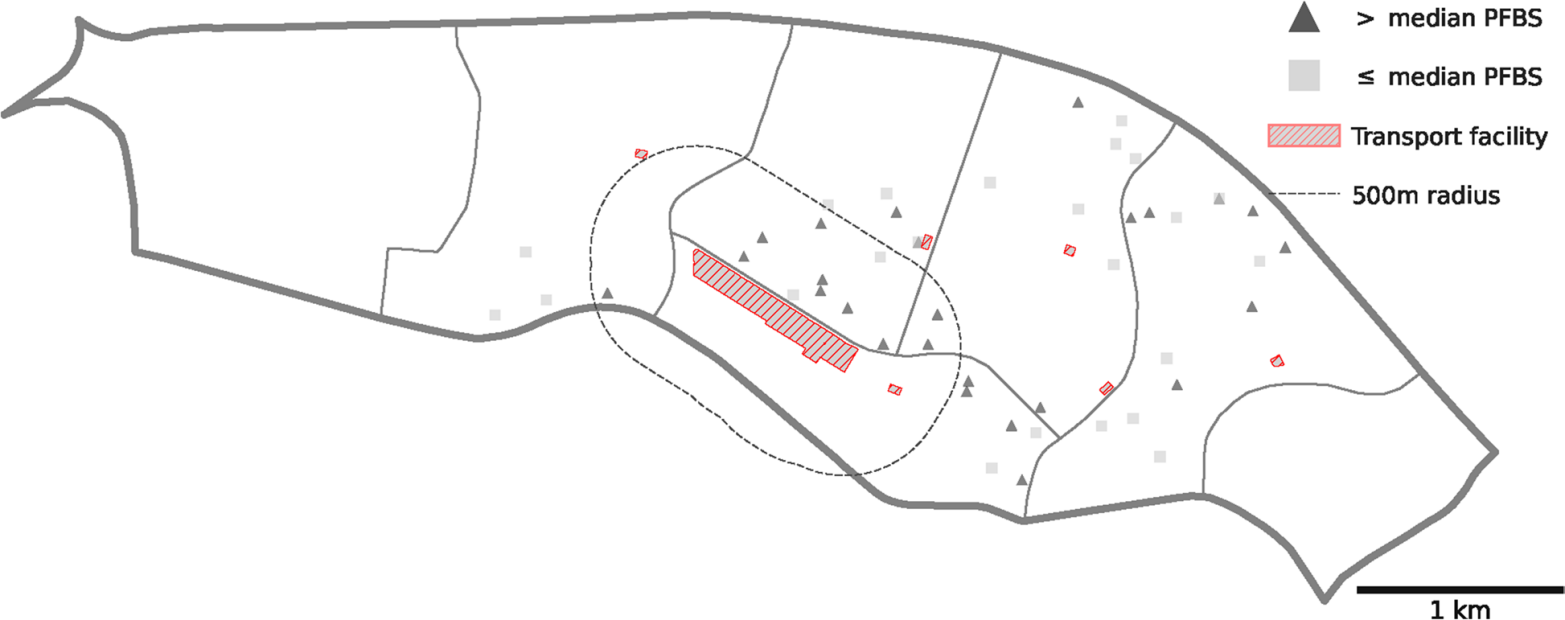
Participants around a transit depot in the area identified in the neighborhood-level influential point analysis. Each triangle and square marker corresponds to a participant living in this area. Lines delineate administrative borders

In dose-response analyses, we found stronger associations with PFBS when considering only transport facilities > 10,000m${}^2$ and inconsistent results for smaller facilities (Fig. 5). Influential point analyses found one neighborhood strongly increased the overall estimate of association (Figures A15 to A16 report individual- and neighborhood-level influential observations from jackknife analyses) with several others showing strong influence. In graphical inspection, large transport depots (> 10,000m${}^2$) exist in these neighborhoods (e.g., see Fig. 6). Individuals residing in neighborhoods with at least 12,000m${}^2$ of transport facilities had 7.3 ng/mL higher plasma PFBS on average ($\hat{\beta}$ = 7.326 ng/ml, SE 3.352, p =.037), while those with at least 20,000m${}^2$ had 11.5 ng/mL higher PFBS on average ($\hat{\beta}$ = 11.530 ng/ml, SE 2.459, p < .001). Similar results are shown for other PFAS, some with a smaller threshold of 4,000m$^2$ (Appendix F.A; Figure F30 reports threshold analyses for all eight PFAS compounds).

### Sensitivity analyses

We found little evidence that transport facilities were strongly correlated with or their effects explained by other land use types (Appendix C; Figure C26 tests associations with all alternative land use classifications). Adding neighborhood-level potential confounders, including other land use types, neighborhood affluence, and urban density levels, confirms that exposure to transport facilities is not just proxying for such factors (Figure A11, Models adjusted for additional built and social environment factors). Alternative spatial measures (Appendix D) and future transport facility exposures using future residence (Appendix E) also did not explain the observed associations. In the S-PRESTO replication analyses, we likewise find statistical evidence of positive associations: a SD increase in transport facilities is associated with 0.10 (0.12 ng/mL), 0.11 (0.075 ng/mL), 0.10 (0.0032 ng/mL), and 0.12 (0.026 ng/mL) SD increases in PFOS, PFNA, PFHpS, and PFHpA, respectively (Appendix F; Figure F31 reports S-PRESTO estimates), however PFBS was not detected in S-PRESTO.

## Discussion

In a demographically-and geographically-representative sample of pregnant women in a dense, urban city, we find robust statistical evidence of a link between residential proximity to large transport facilities, such as bus terminals, and several PFASs quantified in blood. Specifically, we found that a one SD higher exposure to transport facilities land cover was associated with a 0.12 SD increase in PFBS, a 0.17 SD increase in PFBA, and a 0.11 SD increase in PFNA (Fig. 4). Ground-truthing and dose-response analyses suggested that these signals are likely due to the presence of large bus terminals, as opposed to small petrol (gas) stations. Individuals residing in neighborhoods with at least 20,000m$^2$ of bus terminals had an average of 11.5 ng/mL, or nearly ${}^{3}\!/{\!}_{4}$ of an SD, higher plasma PFBS than those who had less exposure to transport facilities.

### Health effects

PFBS and PFBA are newer replacements [1, 62] for legacy PFAS such as PFOA and PFOS with known adverse health effects [1, 2, 8–10, 75–78]. Given their more recent introduction, shorter half-lives, and widely varying toxicokinetic properties [2, 62, 79], PFBS and other shorter-chain PFAS remain understudied. One recent animal model study found PFBS effects on adverse maternal outcomes, including renal injury and placental function [80]. Three human studies using the same sample found links between serum PFBS concentration and asthma, immunological markers, and hyperuricemia [2, 62, 79]. PFNA remains similarly understudied. Mouse studies have found links between PFNA and developmental toxicity [81, 82]. More recent human pregnancy studies have associated PFNA with infertility and low birth weight [83–87], as well as metabolic syndrome in adults [88].

### Established urban determinants of PFAS exposures

Endogenous exposure to environmental chemicals is generally not well studied in Singapore. However, a few studies show higher relative PFAS concentrations in adults living in Southeast and East Asia, relative to elsewhere. Several studies in Singapore have shown relatively high levels of PFAS contamination in environmental samples, especially PFBS in various matrices (e.g., bulk water, suspended particles, and benthic sediments) [19, 44, 67]. A study of urban catchment environmental samples targeting 22 PFAS in Singapore found the most abundant to be PFBS [19], far exceeding other studies [44]. In our study, we support past environmental studies by finding relatively high endogenous PFBS concentrations in pregnant women, compared to other adult populations without occupational exposures [45]. Furthermore, we add to this literature by examining and identifying potential urban built environment determinants, narrowing in on transport-related land use. Past studies have typically evaluated acute contamination events or otherwise regulated or well-known sources such as manufacturing, airports, or military facilities [13, 23, 61, 89, 90].

Interestingly, we found no associations between industrial areas and plasma PFAS (Appendix C), which are known point sources of PFAS [12–15, 17, 90]. These may be attributable to effective regulatory buffers (Figure A1), and therefore indicate a potential policy solution to transit facilities.

### Transport facilities as urban determinants

Few studies have investigated common, unregulated, persistent urban environmental sources. Transport facilities are a high-potential point source of PFAS exposure due to numerous known applications in automotive parts and products [24]. These include auto body paints and top coats, engine and steering system sealants and bearings, pipe linings, engine oil coolers, and liquid storage containers [24]. Importantly, unlike industrial or other land use types, there are typically no restrictions on siting housing near transport facilities. Based on our geospatial computations, residences were as close as 18 meters ($\sim$60 feet) to a transport facility (e.g., see Fig. 6).

Singapore’s unfavorable geology (low-permeability granite substrate) limits groundwater extraction as a drinking water source [91]. Instead, Singapore relies on a centralized water system consisting of rainwater from water catchments, imported water, high-grade reclaimed water, and desalinated seawater. This contrasts with cities where groundwater extraction from contaminated aquifers could serve as a drinking water PFAS source [23]. While high PFAS concentrations have been detected in environmental samples around catchment reservoirs [19], a recent study found low PFAS concentrations in Singapore’s drinking water [92]. However, since water treatment processes may have improved over time and/or some compounds may not be included in monitoring [91–93], we cannot necessarily rule out drinking water as a potential exposure pathway during our study periods (2009–2011 for GUSTO, 2015–2017 for S-PRESTO).

Emerging evidence suggests that airborne PFAS represent an underappreciated exposure route [25–28]. A recent extensive field study demonstrated that PFAS can remobilize into the air, amplifying the risk of atmospheric presence and potential exposure in areas beyond the original point source [39]. Moreover, elevated concentrations of PFAS have been detected in the atmosphere and particulate matter near known sources [35, 40–43]. While no measurements of air samples around transport facilities exist, automotive uses involve numerous PFAS-containing products that could generate airborne emissions [24]. Daily fleet washing uses automotive waxes (which aid spreading and improve water/oil resistance) and interior cleaning products for carpets and seats [24]. Routine maintenance of engine and steering systems involves polymeric PFAS sealants and bearings designed for wide temperature ranges [24], with lubricants and hydraulic fluids containing high PFAS concentrations [94]. These compounds can volatilize and partition between gas and particle phases [32], with higher temperatures (e.g., in Singapore) potentially facilitating volatilization from contaminated surfaces [67]. Atmospheric transport can then disperse these airborne PFAS to nearby residential areas [35, 39], where exposure through inhalation and dust ingestion has been shown to correlate with blood PFAS concentrations [95].

Based on our ground-truthing of transport facilities through site images (Auditing land parcels for transport facilities section), they primarily comprise neighborhood petrol stations and bus depots and terminals. Their sizes vary considerably: petrol kiosks typically have footprints of around 1000–4000m$^2$, while bus depots and terminals are anywhere between 10,000 to over 50,000m$^2$ (Appendix B). Dose-response analysis reveals stronger PFBS associations for exposure areas that exceed 10,000m$^2$, beyond the size of the typical petrol kiosk. These estimates indicate that a small neighborhood petrol kiosk (at least as the only source of cumulative exposure area) is unlikely to be the driver of the observed associations. Instead, larger depots and terminals, where operational intensity (as discussed above) differs substantially from that of smaller facilities, likely drive the observed associations with elevated blood PFAS concentrations in residents.

In the land use sensitivity analyses that consider all other land use types, only the “Business 2 - White” type (designated for mixed industrial/commercial/residential/recreational use) is correlated with both transport facilities and PFAS. Such mixed-use areas include small retail establishments (e.g., vets, cafes), but also include small automotive-related businesses (e.g., car repair workshops), consistent with automotive-related products and activities as possible sources. However, it is unlikely that exposure to transport facilities, especially the larger depots, is simply proxying for such neighborhood-level confounders. When we add exposure to Business 2-White and various neighborhood-level factors (e.g., affluence, urban density), the associations for transport facilities persist (Figure A11).

Dense urban settings make close proximity to transport facilities highly likely events. In our study, the neighborhood with the largest bus terminal facility (Fig. 6) had a population of 30k residents (density = 20k / km$^2$) around the time of the study (2010 Census). As of the latest 2020 census, this neighborhood had increased in density to 32k/km$^2$. On the other hand, transport facilities have remained steady over time: Our computations suggest that total transport facilities changed by less than 0.03% between 2008 and 2019 for areas within a 500m radius of known public housing blocks. These trends suggest that urban PFAS exposures due to transport land use will only intensify with time.

### Cohort variation in spatial associations

The spatial associations to differing congeners in the GUSTO ($\sim$2010; PFBS/PFBA/PFNA) and S-PRESTO ($\sim$2016; PFOS/PFHpS/PFHpA/PFNA) cohorts warrant attention.

To our knowledge, the only PFAS regulated under Singapore’s Environmental Protection and Management Act since 1999, including its revisions, are PFOA, PFOS, PFHxS [96]. Recent phase-out of certain fire-fighting foams (announced 2024, with effect from 2026) is likewise limited to PFOA, PFOS, and PFHxS [97]. Hence, PFBS and most other PFAS remained unregulated during both sampling periods. That said, PFASs have received increased attention in Singapore with the national water agency (PUB) beginning routine monitoring of PFAS in drinking water in 2016 [93].

Internationally, PFBS was added to the EU REACH Candidate List of Substances of Very High Concern in 2020 for “probable serious effects to human health” [98]. Consequently, Singapore may have begun monitoring and treating for PFBS and other PFAS around the time of the second cohort’s enrollment (2015-2017). However, we are not aware of any official documentation of specific compounds tested [93]. Additionally, manufacturers may be voluntarily changing formulations of products to remove congeners such as PFBS in anticipation of pending action by the EU and internationally.

While older studies of groundwater and reservoirs detected PFBS, no recent studies have documented trends or the presence of PFBS in drinking water or other exposure routes [19, 44, 67]. Based on a government report in 2022 and a study in 2025, there appear to be negligible levels of PFAS in drinking water [92, 93]. However, those reports follow well after our study period. Other studies in the region suggest increasing and changing patterns of airborne (suspended) PFAS, particularly in coastal regions. Notably, PFBS and PFBA are found at relatively high proportions in suspended PFAS [99]. A final potential source of secular trends is in food contamination, as nearly all of Singapore’s food is imported. Due to groundwater and pesticide contamination, PFASs are highly detected in crops from regional suppliers [100]. Importantly, short-chain PFAS are more readily taken up by plants [101]. That said, there have been no official studies or statements on PFAS food contamination from the relevant oversight body (Singapore Food Authority) that we are aware of.

We also note that the timing of sampling and the biological matrices vary slightly across cohorts. For GUSTO, we examined cord blood plasma collected at delivery. By contrast, for S-PRESTO, we examined womens blood plasma collected at enrollment prior to conception. Cord blood reflects fetal PFAS concentrations resulting from placental transfer of cumulative maternal burden over the gestational period, while plasma reflects direct maternal burden. While the near-absence of detectable PFBS in S-PRESTO (1 of 384 samples) compared with universal detection in GUSTO is unlikely to be fully explained by physiological changes in pregnancy, we note that we and others have shown that shorter-chained PFAS preferentially partition across the placenta [102]. Unfortunately, we did not have paired cord blood samples for S-PRESTO to investigate this directly.

Consequently, as the first study to investigate trends in PFAS exposures and their relation to the built environment in Singapore, we cannot definitively disentangle whether differences in association reflect secular trends in PFAS sources, exposures, or differences in biological matrices. Future studies are needed to replicate and confirm these trends and rule out alternative explanations. Importantly, direct assessment of suspended airborne PFAS, especially near bus terminals, is warranted.

### Strengths and limitations

That said, correlations between proximity to putative contamination sources and endogenous PFAS concentrations are not sufficient to pinpoint sources. Notably, spurious correlations can arise if certain neighborhoods are over-represented by individuals with higher (or lower) relative PFAS exposure through other exposure routes such as diet or other exposure-related behaviors. For example, individuals who are particularly conscious about exposure to air and sound pollution may choose not to live near transport facilities. Our study is strengthened by the large array of sensitivity tests aimed at resolving these alternative explanations and the potential for spurious findings. Most notably, we rule out correlations with other types of land use (Appendix C), directly adjust for potential environmental confounders (Figure A11) and neighborhood to account for the myriad unmeasured factors which may determine PFAS exposure, such as drinking water sources and proximity to other sources of exposure. We also exclude the possibility that personal behavioral choices may explain observed observations, by examining associations with future residential exposure as a placebo or negative control exposure. In finding no association between PFAS concentration in pregnancy and future residential exposures (Appendix E), we rule out individuals’ preferences or choices as the reason for associations (rather than the exposure to transport facilities itself). Finally, we replicated our findings in a separate cohort (Appendix F), albeit with different PFASs than the original cohort.

We note several limitations to our study. First, given our sample and context of analyses, we cannot precisely characterize whether pathways occur indoors at home or outdoors around the neighborhood. Previous studies have highlighted indoor air pollutants, potentially through heating, ventilation, and air conditioning systems [29, 33, 59]. We are cognizant that occupation is an important determinant of PFAS exposure, and we adjust for it. However, the cohort occupation classification may not align directly with related occupational hazards [23, 49, 56–58]. Not all PFAS are well detected, and we only report PFAS that were well detected. Notably, PFBS were not well detected in our S-PRESTO cohort, highlighting the challenge of detecting chemicals with relatively shorter half-lives. A related issue is that while PFAS are often present as mixtures, we study only a few in isolation [77]. Future studies should examine spatial determinants of mixtures. Finally, we lack concrete priors about the exposure to transport facilities and thus modeled it linearly with the primary evaluation buffer at 500 meters. The upshot is that we find consistent evidence across different-sized buffer model specifications.

## Conclusions

More than half of the world’s population lives in urban areas, with urbanization continuing to accelerate [103]. This study addresses a critical research gap in understanding common, unregulated urban determinants of PFASs, which may have wide-ranging adverse health effects, especially during pregnancy. We find robust evidence that transport facilities may be an otherwise unrecognized source of PFAS exposures. Singapore has lower geographical disparities in poverty and minority status, reducing the likelihood of confounding in our study. However, the corollary is that our findings may represent a lower boundary on PFAS determinants as compared to other regions with greater sociodemographic segregation and issues of environmental injustice. Since transport facilities are much more prevalent than, e.g., factories or airports in urban areas, the contribution of differential exposure of PFAS and other understudied chemicals to geographic health disparities from these common built environment features warrants far more attention.

## Supplementary Information

Supplementary Material 1.

## Data Availability

The data that support the findings in this study are available upon reasonable request to the GUSTO Executive Committee. The procedure for requesting data can be found here: https://gustodatavault.sg/about/request-for-data. Code to reproduce results is publicly available at https://github.com/lsys/tfpfas.
