# Supplementary material for: Residential proximity to transport facilities as urban determinants of individual-level per- and poly-fluoroalkyl substance (PFAS) exposures: Analysis of two longitudinal cohorts in Singapore
Source: Environ Health. 2026 Jan 9;25:32. doi: 10.1186/s12940-025-01257-5 (PMC13069709; doi:10.1186/s12940-025-01257-5)
Supplement: Supplementary file 1 — Supplementary Material 1. [file 12940_2025_1257_MOESM1_ESM.pdf]

Supplementary Materials for
“Residential proximity to transport facilities as urban determinants of individual-level per- and poly-fluoroalkyl substance (PFAS) exposures: Analysis of two longitudinal cohorts in Singapore”
(December 2025)

Table of Contents

§A: Supplementary materials for results

§B: Public-contributed street-level pictures of transport facilities

§C: Other land uses

§D: Other spatial instruments

§E: Movers and future exposure

§F: Other PFAS

A. Supplementary materials for results

A.A Descriptive and summary results

This appendix provides supporting tables, figures, and details for descriptive results. All distributions, including spatial distribution of the land parcels of interest, are reported here.

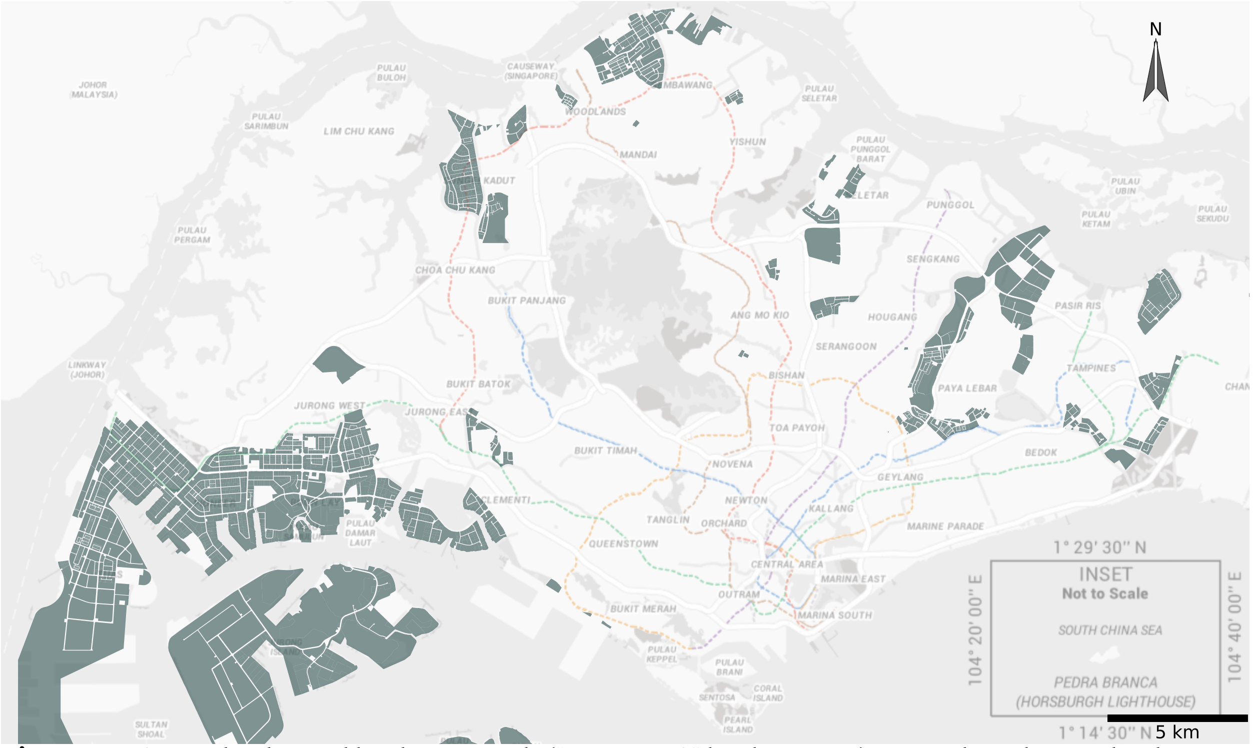

Figure A1. General industrial land use parcels (“Business 2” land use type). From the Urban Redevelopment Authority’s Master Plan 2008

Figure A1 plots the land parcels most closely related to heavy industrial sites (Urban Redevelopment Agency "Business 2" land use). Most are scattered on the outskirts of residential areas, in particular the large parcels. A minority of smaller land parcels classified as "Business 2" are near or within neighborhoods (referenced in https://www.ura.gov.sg/-/media/Corporate/Planning/Master-Plan/MP19writtenstatement.pdf).

In auxiliary descriptive analyses, we find correlations between the plasma PFBS concentrations and a set of maternal factors. All findings below are correlations of the data without any adjustments, unless reported otherwise. We find that low-income mothers (less than SGD2000 in monthly income; 67.3%; n = 739) have higher PFBS measurements. Mothers without college degree (73.1%, n = 772) have higher PFBS measurements. Mothers living in public housing (90.7%; n = 772)—which corresponds to more affordable housing from government housing subsidies—have higher PFBS measurements: Living in public housing is associated with approximately 4.01 ng/ml (SE = 1.63; $p < .05$) higher plasma PFBS concentration. This association persists even after adjusting for maternal baselines and attenuates to null only when we include the neighborhoods fixed effects.

Correlation of GUSTO participants with base population. While GUSTO was not designed to be geographically representative,[101] we find that our sample of GUSTO participants closely follows the geographical distribution of the base population–women aged 20–49. We start by retrieving the Department of Statistics population census numbers (https://www.singstat.gov.sg/find-data/search-by-theme/population/geographic-distribution) for the 2010 census which is available by planning areas and subzones (*neighborhoods*). The participant age range at delivery is approximately 19–47. We therefore filter the population census figures for females who fall in the six relevant 5-year age bins (age 20–24, age 25–29, age 30–34, age 35–39, age 40–44, age 45–49). We then groundtruthed the official subzone of each GUSTO participant by first geocoding their location using the postal code before map-matching their point locations to the official 2008 version of the subzone delineation map. This yields a subzone (and planning area) ID tag for each GUSTO participant which we use to match to the subzone tags in the population census figures. Finally, we compute the correlation between the number of GUSTO participants and the number of women aged 20–49 in the base population (planning area correlation = .895, p-value $< .001 \times 10^{-13}$, planning areas = 33; subzone correlation = .89, p-value $< .001 \times 10^{-58}$, subzones = 166; GUSTO participants = 1,488).

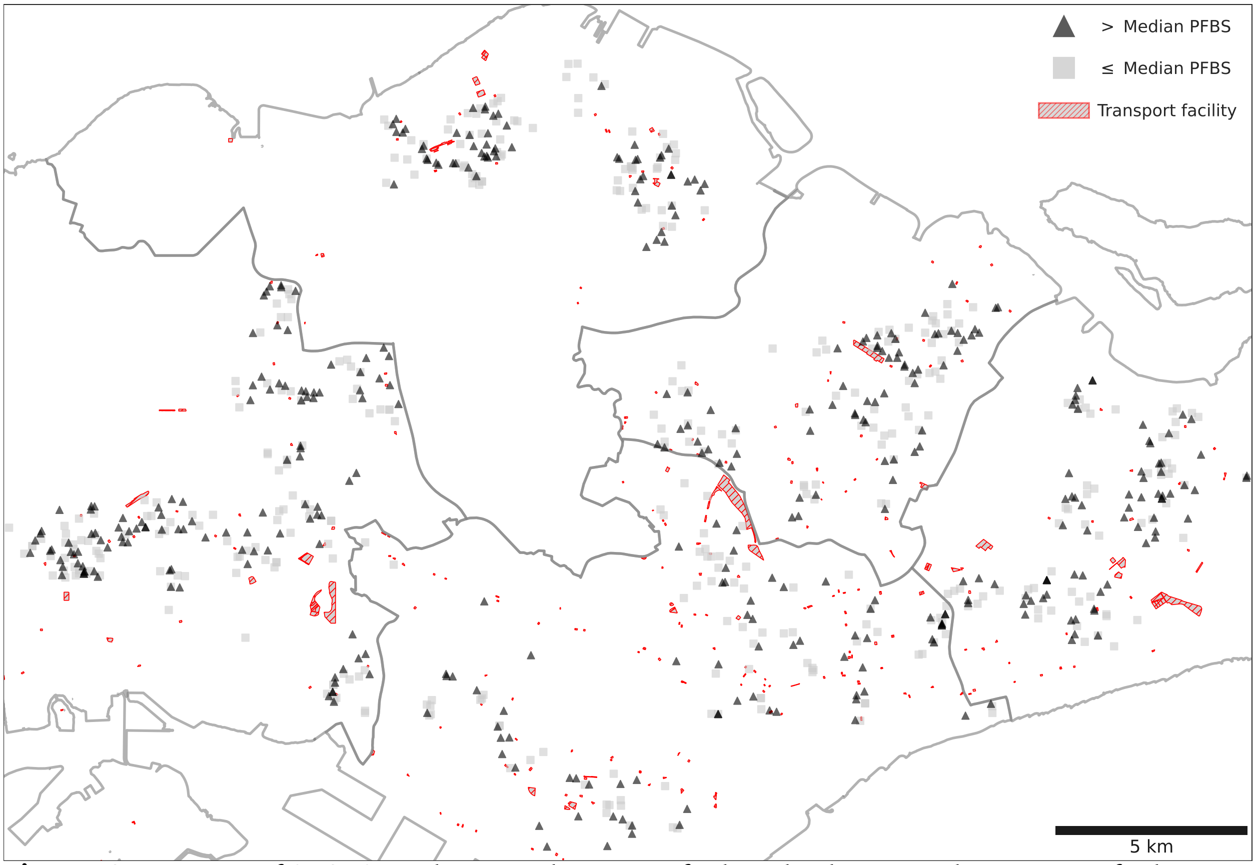

Figure A2. Locations of GUSTO residences and transport facilities land use parcels. Transport facilities are shaded in red. Gray lines delineate the five regions (central, west, east, north, north-east). See Figures A3 to A7 for a breakdown of the same figure by the other five regions.

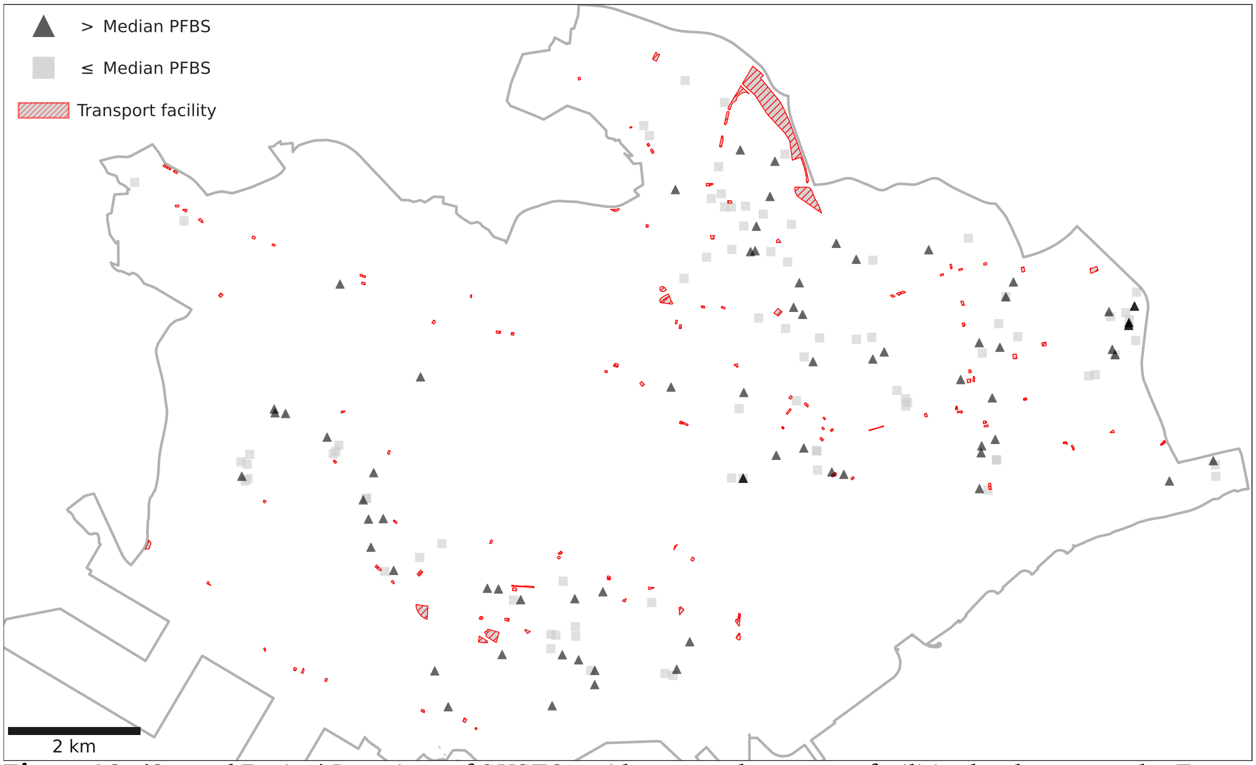

Figure A3. (Central Region) Locations of GUSTO residences and transport facilities land use parcels. Transport facilities are shaded in red. GUSTO residences are in triangles (above median plasma PFBS concentration) and squares (below median plasma PFBS concentrations). See Figure A2 for the map of the whole city. See Figures A4 to A7 for the other four regions.

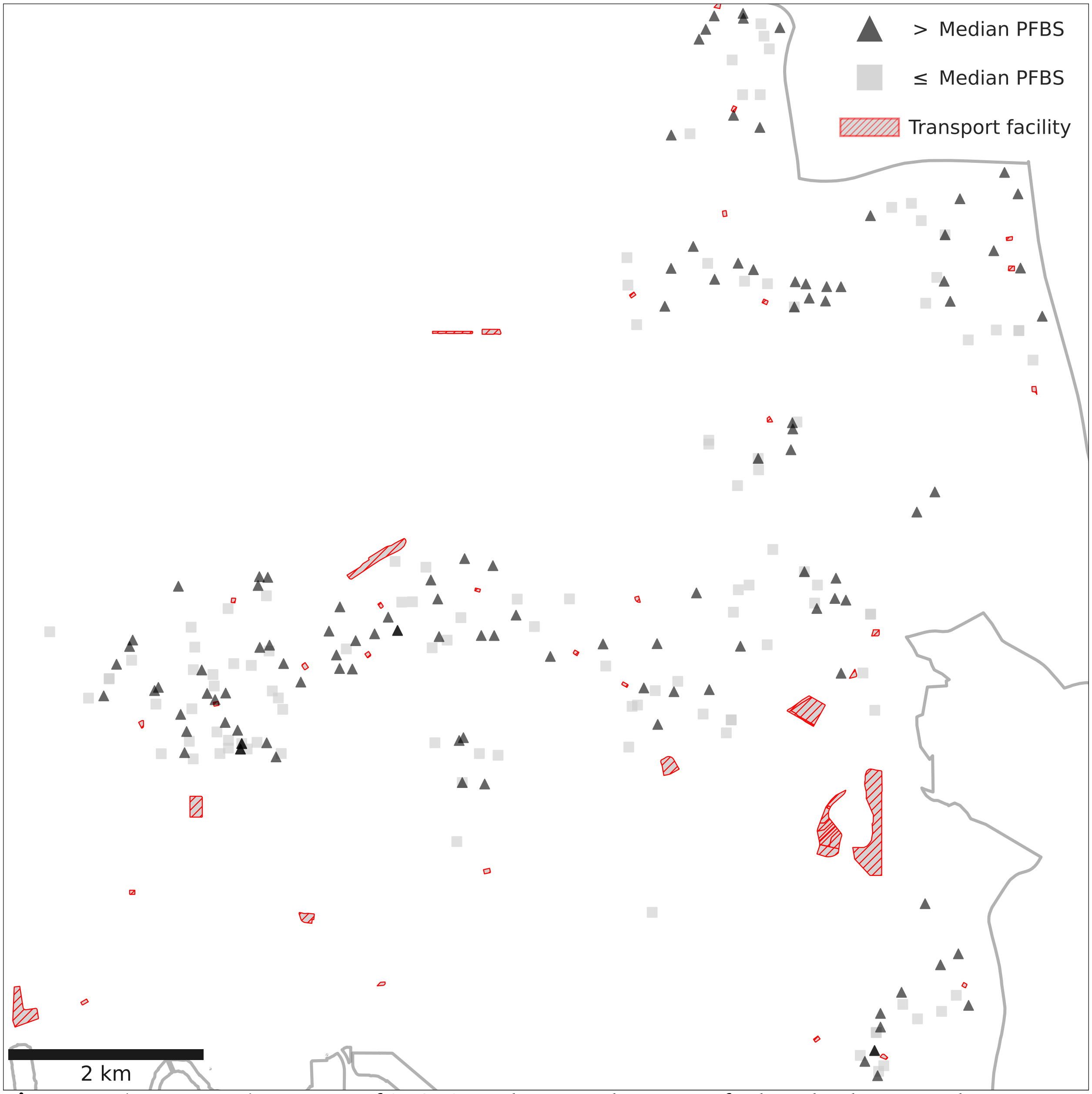

Figure A4. (West Region) Locations of GUSTO residences and transport facilities land use parcels. Transport facilities are shaded in red. GUSTO residences are in triangles (above median plasma PFBS concentration) and squares (below median plasma PFBS concentrations). See Figure A2 for all five regions.

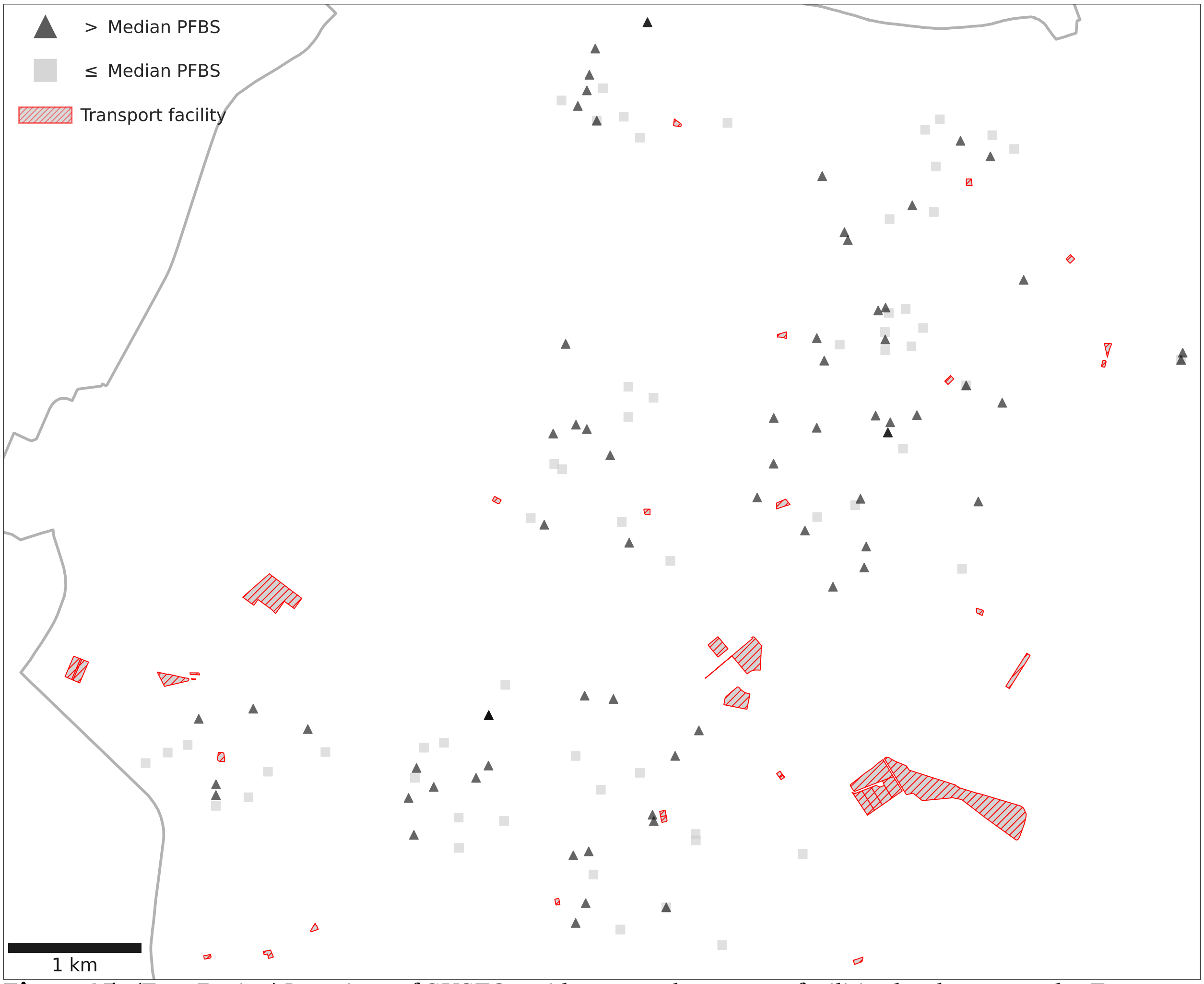

Figure A5. (East Region) Locations of GUSTO residences and transport facilities land use parcels. Transport facilities are shaded in red. GUSTO residences are in triangles (above median plasma PFBS concentration) and squares (below median plasma PFBS concentrations). See Figure A2 for all five regions.

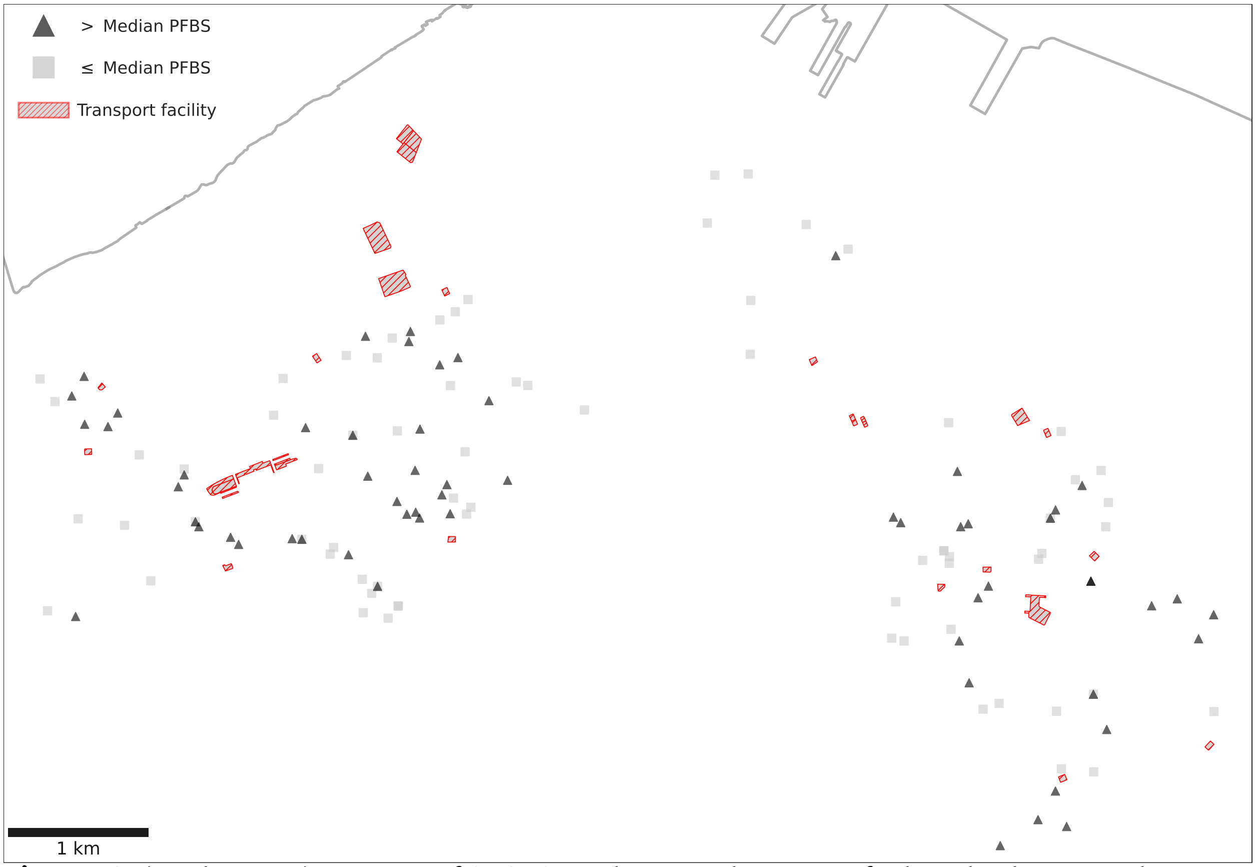

Figure A6. (North Region) Locations of GUSTO residences and transport facilities land use parcels. Transport facilities are shaded in red. GUSTO residences are in triangles (above median plasma PFBS concentration) and squares (below median plasma PFBS concentrations). See Figure A2 for all five regions.

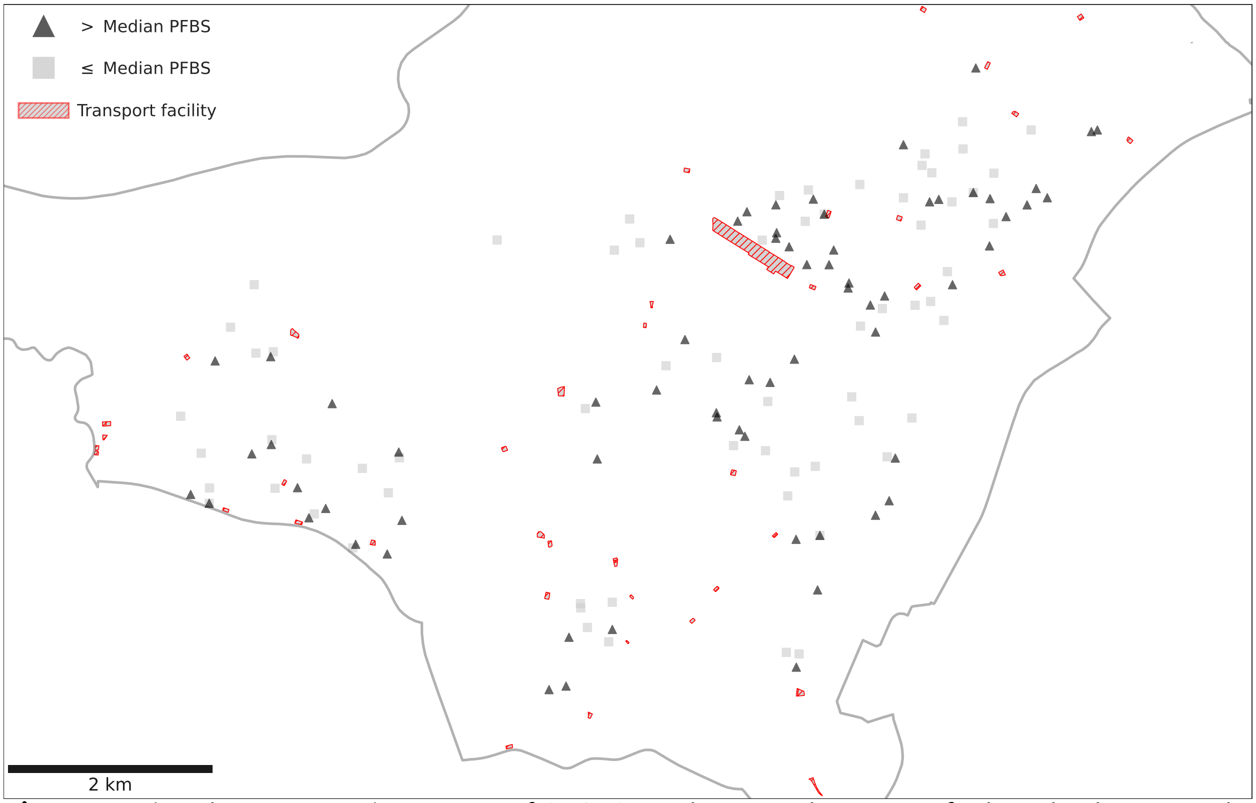

Figure A7. (North-East Region) Locations of GUSTO residences and transport facilities land use parcels. Transport facilities are shaded in red. GUSTO residences are in triangles (above median plasma PFBS concentration) and squares (below median plasma PFBS concentrations). See Figure A2 for all five regions.

Table A1. Summary of GUSTO participants

	Category	N	%
Age at delivery	Mean/SD	30.5	5.3
Ethnicity	Chinese	370	48.1%
	Malay	240	31.2%
	Indian	159	20.6%
Place of birth	Singapore	485	64.5%
	China	90	12.0%
	Malaysia	81	10.8%
	India	63	8.4%
	Indonesia	18	2.4%
	Bangladesh	6	0.8%
	SriLanka	4	0.5%
Highest education level	None	2	0.3%
	Primary	47	6.2%
	Secondary	219	28.9%
	General Certificate of Education	199	26.2%
	Institute of Technical Education	90	11.9%
	College	202	26.6%
Occupation	Homemaker	207	27.6%
	Professional	166	22.1%
	Clerical Worker	128	17.0%
	Service Worker	102	13.6%
	Technician Associated Professional	80	10.7%
	Legislator Senior Official	20	2.7%
	Unemployed	16	2.1%
	Student	13	1.7%
	Plant Machine Operator	12	1.6%
	Others	5	0.7%
Public/private housing	Public housing	688	90.6%
	Private housing	71	9.4%
Marital status	Married living with husband	717	95.5%
	Single not living with baby father	17	2.3%
	Single living with baby father	14	1.9%
	Married not living with husband	2	0.3%
Monthly income	0–999	258	35.5%
	1000–1999	233	32.0%
	2000–3999	189	26.0%
	4000–5999	37	5.1%
	More Than 6000	10	1.4%

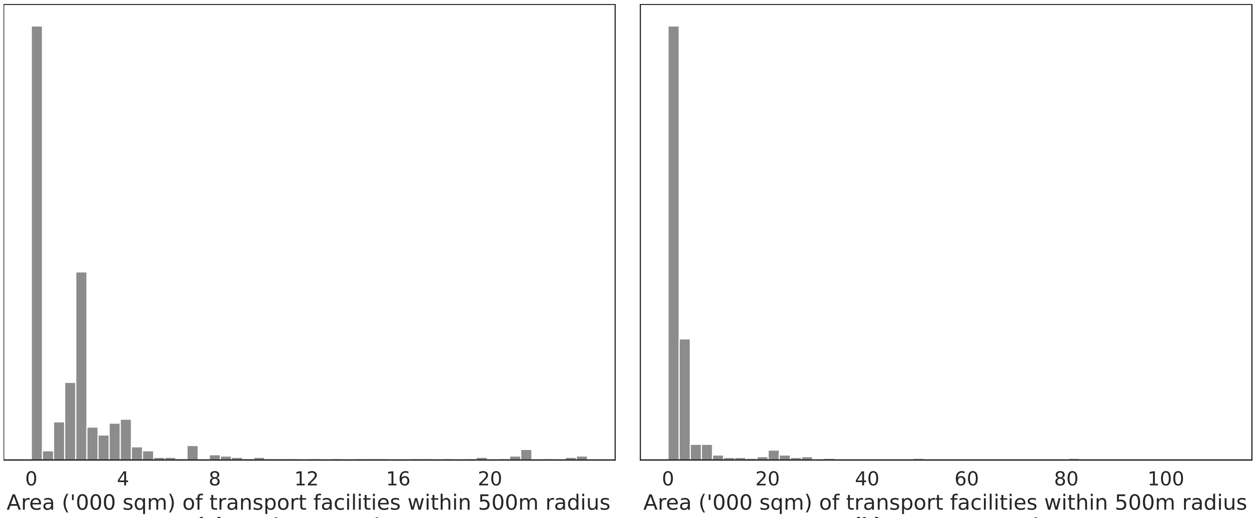

(a) Without outliers **(b)** Untruncated

Figure A8. Distribution of area of transport facilities land parcel within 500m radius of residence. Vertical axis is density. Left figure cuts off at approximately 97th percentile. See Figure A9 for the distribution based on a 1000m radius.

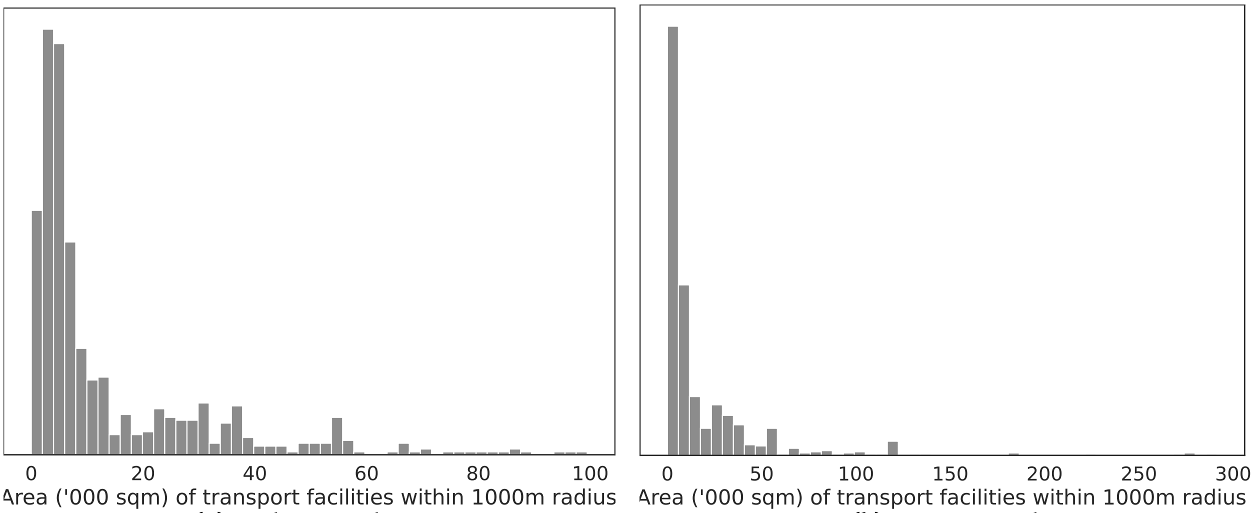

(a) Without outliers **(b)** Untruncated

Figure A9. Distribution of area of transport facilities land parcel within 1000m radius of residence. Vertical axis is density. Left figure cuts off at approximately 97th percentile. See Figure A8 for the distribution based on a 500m radius.

Table A2. Completeness of GUSTO EDC measures

	Measured	Not measured			Summary statistics				
	Measured (1)	< LOD (2)	< LOQ (3)	Total (4)	Mean (5)	SD (6)	Q1 (7)	Median (8)	Q3 (9)
PFBS	784	0	0	0	24.162	15.929	13.28	18.23	28.95
MBP	784	0	0	0	2.71	1.61	1.75	2.29	3.122
PFOA	784	0	0	0	1.982	1.544	1.0	1.485	2.415
PFNA	784	0	0	0	0.951	0.528	0.59	0.79	1.212
MEHP	784	0	0	0	10.712	7.614	6.77	9.065	12.24
Oxyben	784	0	0	0	2.269	0.784	1.77	2.11	2.68
PFOS	783	0	1	1	1.326	0.986	0.77	1.11	1.58
PFHxS	777	1	6	7	0.579	0.359	0.33	0.5	0.73
Benzophe	775	9	0	9	57.943	157.356	2.74	6.89	39.045
PFBA	773	11	0	11	2.076	4.34	1.25	1.58	2.06
PFUnDA	754	2	28	30	0.256	0.222	0.14	0.2	0.29
PFDA	750	0	34	34	0.162	0.1	0.11	0.14	0.19
Methylp	663	119	2	121	6.889	7.171	2.39	5.47	9.1
MECPP	655	129	0	129	0.597	0.615	0.29	0.42	0.685
MEP	558	209	17	226	1.93	3.335	0.582	0.9	1.725
PFHpA	555	139	90	229	0.196	0.169	0.11	0.15	0.21
MMP	526	258	0	258	0.911	1.469	0.61	0.75	0.93
MCINP	515	269	0	269	2.972	2.994	1.41	2.15	3.32
Propylp	506	278	0	278	0.366	0.437	0.19	0.25	0.39
BPS	437	326	21	347	0.179	0.22	0.09	0.12	0.19
Ethylp	388	359	37	396	0.26	0.245	0.13	0.2	0.29
PFPeA	207	577	0	577	0.175	0.117	0.12	0.15	0.19
PFDoDA	82	139	563	702	0.109	0.096	0.07	0.09	0.11
BPA	58	726	0	726	10.544	17.001	3.885	5.315	9.608
Butylp	11	756	17	773	0.125	0.084	0.07	0.1	0.13
MCPP	10	661	113	774	0.583	0.411	0.375	0.425	0.488
MBzP	9	773	2	775	1.477	0.756	0.81	1.35	1.85
PFHxA	7	776	1	777	0.933	0.449	0.64	0.85	1.195
MNOP	0	784	0	784	—	—	—	—	—

Note: Table reports the completeness of PFAS measurements. Measurements in nanograms per millilitre (ng/ML). LOD = limit of detection. LOQ = limit of quantification. Samples flagged as < LOD and < LOQ are imputed with the corresponding LOD/LOQ values divided by $\sqrt{2}$ in Appendix F.

Table A3. Eight types of transport facilities

**Facility Type**
Car Park
Heavy Vehicle Park
Trailer Park
Bus Depot/Terminal
Transport Depot
MRT/LRT Marshalling Yard/Depot
Driving Circuit/Test Centre
Petrol Station/Kiosk

Type of establishment in transport facilities land use parcels. Official source: https://web.archive.org/web/20230207075239/https://www.ura.gov.sg/maps/media/mp/MPUG_2014_for_map_legend.htm.

A.B Main results

This appendix supplements figures and tables for the main results where we evaluate the adjusted associations between plasma PFBS concentrations with exposure to transport facilities. Models are as described in Section 2.7 of the main body.

Table A4 reports the estimates from modeling the plasma PFBS measurements as dependent on exposure to transport facilities.

We have GUSTO 784 participants with blood samples sent for PFAS analysis. 13 participants reported postal codes not found from an official source (https://www.onemap.gov.sg/apidocs/apidocs/#search). These 13 participants drop out in Model 1 of Table A4. In Model 2, 22 participants drop because of missing maternal baselines and 9 because of singletons. In Model 3, there are 30 singletons. A further 50 participants dropped out in our last model, Model 3, when the income measures with lower coverage were included.

Figures A15 to A17 report the results from evaluating which points are influential using the change in the estimated coefficients when certain observations are excluded from estimation. As before, we focus on Model 2. Figure A15 reports the results where each point corresponds to the change in estimated coefficient from Model 2 when the observation indexed on the horizontal axis is omitted from the estimation. The rule of thumb we use is to flag runs where the change is larger than $^2/\sqrt{n}$ in absolute terms. More influential points increase than decrease the estimate. One influential point decreases the estimate by more than half the standardized error in the estimate. In Figure A16, we repeat a similar analysis but omit entire groups of individual-level observation by the subzone they are residing in. We detect one influential subzone from this analysis. We visually analyze this subzone in Figure 6. Figure A17 repeats the above analysis for the broader planning areas. We detect no influential planning areas.

Figure A18 provides three illustrating examples of participants from our GUSTO sample living in the east region. Two participants have exposure to transport facilities, and one does not, as defined using a 500-meter radius using the exposure defined in Section 2.3. The inset in Figure A18 shows the actual gas station identified in the image. The participant with no exposure has a plasma PFBS measurement of 16.93 ng/ml. Based on our estimated (adjusted) coefficient of 0.153 (from Model 2 of Table A4) and the exposure measure of 4.03, together, imply that with all else equal, there would be an estimated 0.62 ng/ml increase in plasma PFBS. In reality, the plasma PFBS concentrations for the two participants with exposure to transport facilities are 18.42 and 23.05, which is slightly higher (by 5% and by 31%, respectively).

Figure A19 provides another illustrating example with a separate pair of participants from the west region. The inset in Figure A19 showing the large transport facility, a bus depot, also shows a heavy industrial area in the backdrop, underscoring the importance of evaluating within-neighborhood variation instead of across-neighborhood variation in our preferred model. The participant in red has exposure to a large transport facility

land parcel, while the other participant in gray square has exposure to a smaller transport facility. This latter participant has a plasma PFBS concentration of 11.27 ng/ml. Based on our estimated (adjusted) coefficient of 0.153 (from Model 2 of Table A4) and the difference in exposure measure of 12.65 (14.653 - 2.003), together, imply that with all else equal, there would be an estimated 1.935 ng/ml increase in plasma PFBS. In reality, the difference in PFBS for these two participants is slightly lower at 1.83.

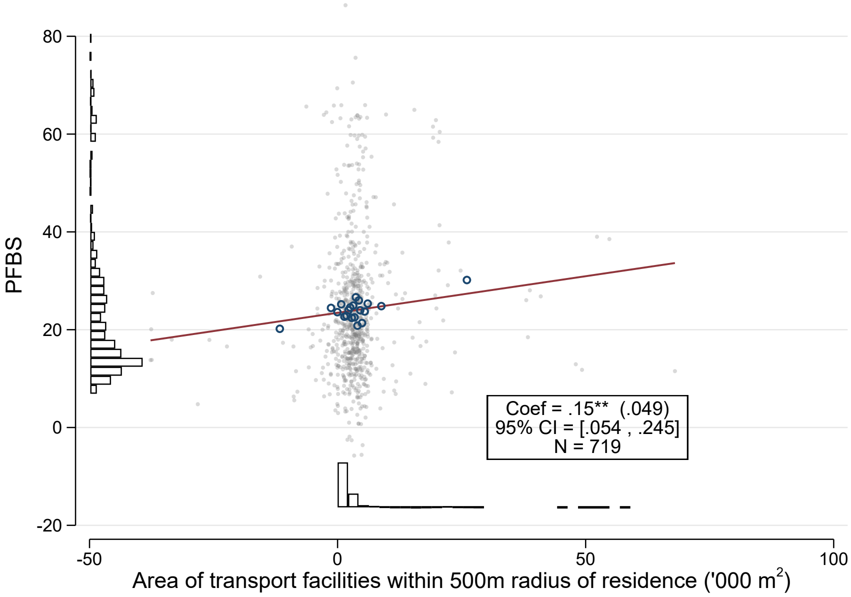

Figure A10. PFBS and transport facilities. See also Figure A8 for the distribution of area of transport facilities. Corresponds to column (2) of Table A4. Standard errors clustered at planning areas. Binned data in blue hollow circles. Significance levels: + 0.1 * 0.05 ** 0.01 *** 0.001.

Table A4. PFBS and transport facilities within 500m radius of residence

	Dependent variable is PFBS (ng/mL)		
	(1)	(2)	(3)
Transport facilities (area) within 500m buffer	0.128^b	0.150^b	0.142^b
	(0.059)	(0.055)	(0.056)
	[0.008–0.248]	[0.038–0.261]	[0.028–0.257]
	$< p = 0.037 >$	$< p = 0.011 >$	$< p = 0.016 >$
Constant	12.779	23.738	19.143
	(17.667)	(22.681)	(28.805)
	[−23.410–48.967]	[−22.722–70.198]	[−39.960–78.247]
	$< p = 0.475 >$	$< p = 0.304 >$	$< p = 0.512 >$
R^2	0.0989	0.195	0.217
Maternal baselines	Yes	Yes	Yes
Income			Yes
Area fixed effects: Planning area	Yes		
Area fixed effects: Subzone		Yes	Yes
Mean Dep Var.	24.1	24.0	23.7
Std. dev. of X	10.3	10.3	10.6
n(Transport facilities (area) within 500m buffer > 0)	438	425	398
n(Clusters)	29	29	28
N	740	719	669

Note: Transport facilities area within 500m buffer is in 1,000 square meters. See Section 2.5 for the maternal and income controls. Figure A10 shows Model 2. Standard errors are clustered at the planning areas. Significance levels: ^{c} 0.1 ^{b} 0.05 ^{a} 0.01.

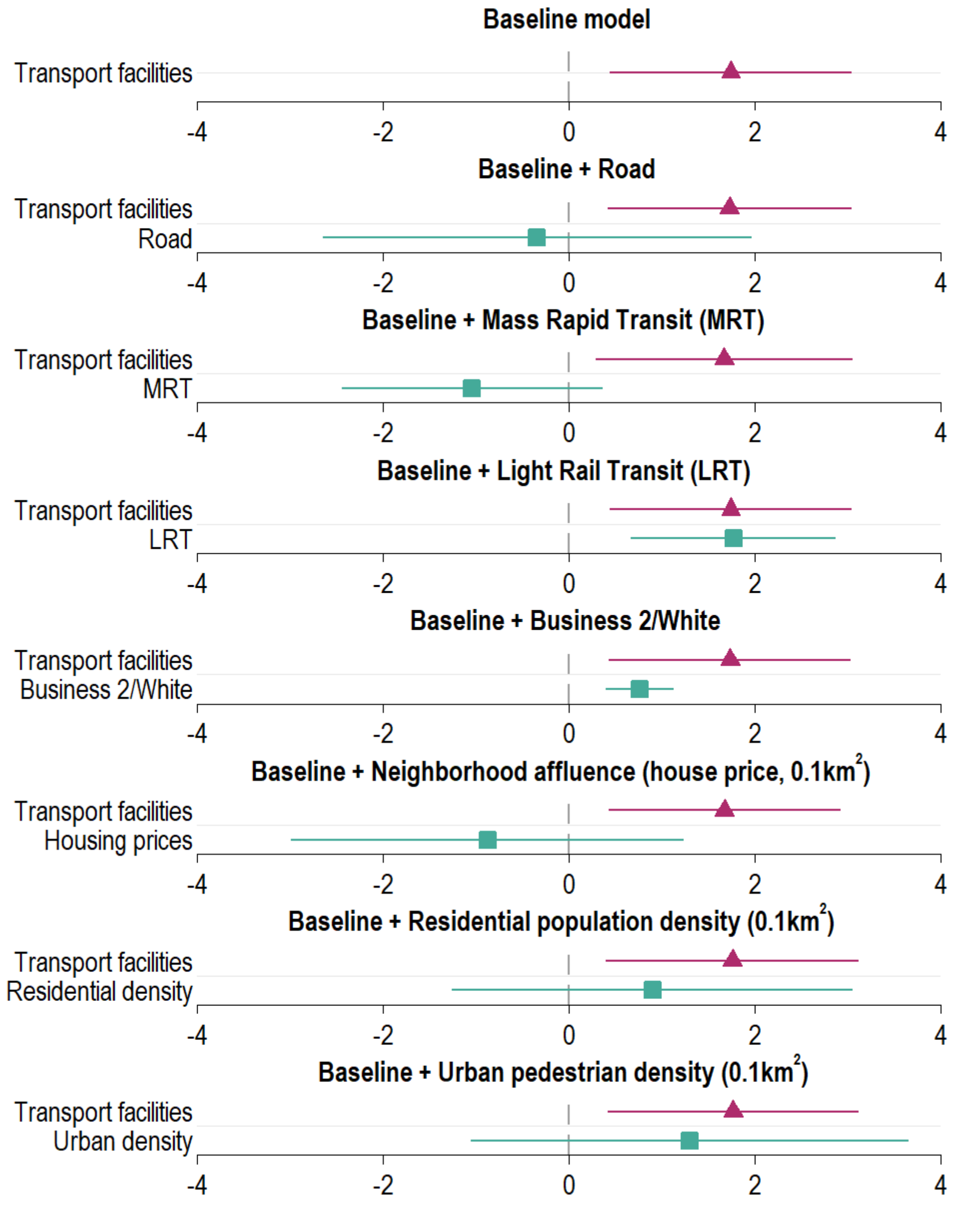

Figure A11. Models with additional environmental covariates. Each model uses PFBS concentration as the outcome, and all coefficients are standardized. Each row shows the coefficient for the area of transport facility within 500 m (red triangles) and the coefficient for the additional covariate (green squares). Starting with the baseline model (top), each subsequent model adds one neighborhood or built environmental characteristic: area of roads within 500 m, area of MRT within 500 m, area of LRT within 500 m, area of Business 2/White (industrial, commercial, residential, recreation mix) within 500 m (Figure C26), neighborhood affluence from housing resale microtransactions at the level of 0.1 km^2,[67] residential population density from the 2010 census smoothed to 0.1 km^2 bins, and pedestrian density from anonymized mobile phone traces smoothed to 0.1 km^2 spatial bins.[67–69]

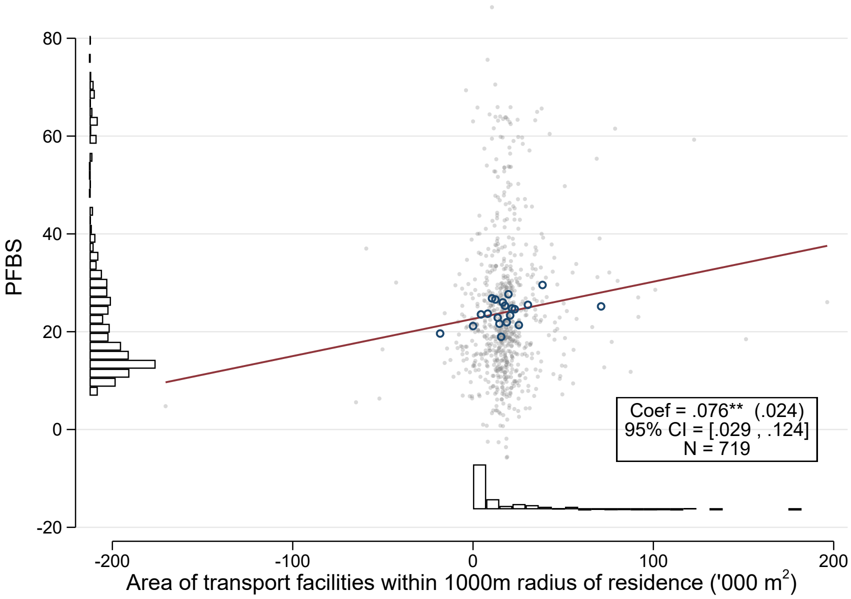

Figure A12. PFBS and Transport Facilities area within 1000m radius. Reported estimates are from regressing PFBS on the area (1,000) of transport facilities within 1000m radius of residence, with subzone fixed effects and maternal characteristics partialed out. Model corresponds to column (2) of Table A5. Standard errors clustered at subzones. Raw data in small gray circles. Binned data in blue hollow circles. Fitted line from the estimated model. Marginal histograms indicate the underlying distributions. Significance levels: + 0.1 * 0.05 ** 0.01 *** 0.001.

Table A5. PFBS and transport facilities within 1000m radius of residence

	Dependent variable is PFBS (ng/mL)		
	(1)	(2)	(3)
Transport facilities (area) within 1000m buffer	0.045^b	0.076^a	0.073^a
	(0.019)	(0.027)	(0.025)
	[0.007–0.083]	[0.021–0.132]	[0.022–0.124]
	$< p = 0.022 >$	$< p = 0.009 >$	$< p = 0.007 >$
Constant	14.664	27.030	22.385
	(17.964)	(23.154)	(29.060)
	[−22.134–51.462]	[−20.398–74.458]	[−37.242–82.012]
	$< p = 0.421 >$	$< p = 0.253 >$	$< p = 0.448 >$
R^2	0.0986	0.199	0.221
Maternal baselines	Yes	Yes	Yes
Income			Yes
Area fixed effects: Planning area	Yes		
Area fixed effects: Subzone		Yes	Yes
Mean Dep Var.	24.1	24.0	23.7
Std. dev. of X	31.8	32.1	32.7
n(Transport facilities (area) within 1000m buffer > 0)	693	677	630
n(Clusters)	29	29	28
N	740	719	669

Note: Transport facilities area within 1000m buffer is the area (in 1,000 square meters) of the buffer area residence that is allocated to transport facilities land use. Figure A12 shows Model 2. Standard errors in (2)–(6) clustered at subzones. Significance levels: ^{c} 0.1 ^{b} 0.05 ^{a} 0.01.

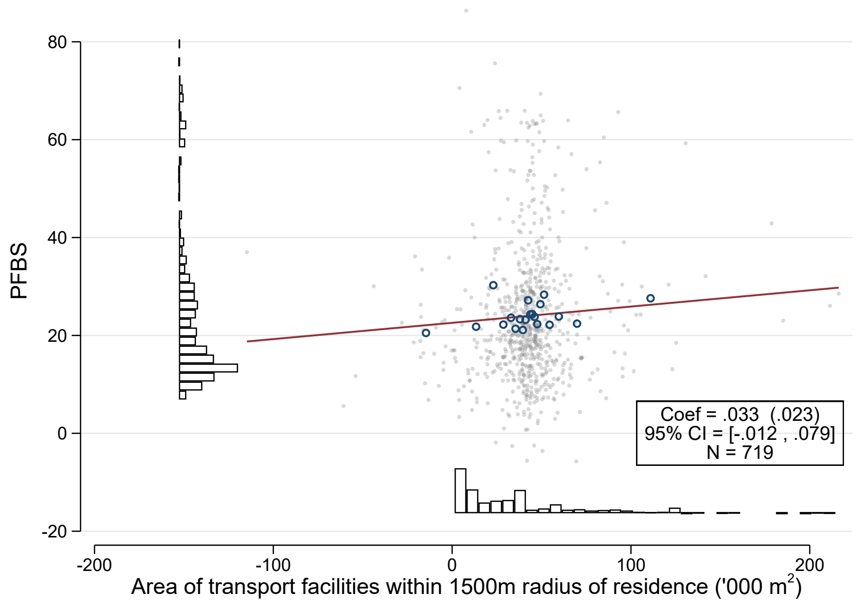

Figure A13. PFBS and Transport Facilities area within 1500m radius. Reported estimates are from regressing PFBS on the area (1,000) of transport facilities within 1500m radius of residence, with subzone fixed effects and maternal characteristics partialed out. Model corresponds to column (2) of Table A6. Standard errors clustered at subzones. Raw data in small gray circles. Binned data in blue hollow circles. Fitted line from the estimated model. Marginal histograms indicate the underlying distributions. Significance levels: + 0.1 * 0.05 ** 0.01 *** 0.001.

Table A6. PFBS and transport facilities within 1500m radius of residence

	Dependent variable is PFBS (ng/mL)		
	(1)	(2)	(3)
Transport facilities (area) within 1500m buffer	0.018	0.033	0.029
	(0.016)	(0.026)	(0.026)
	[−0.015–0.051]	[−0.020–0.086]	[−0.024–0.083]
	$< p = 0.273 >$	$< p = 0.211 >$	$< p = 0.269 >$
Constant	14.086	25.566	20.999
	(17.709)	(23.013)	(28.642)
	[−22.189–50.362]	[−21.574–72.706]	[−37.769–79.766]
	$< p = 0.433 >$	$< p = 0.276 >$	$< p = 0.470 >$
R^2	0.0955	0.193	0.215
Maternal baselines	Yes	Yes	Yes
Income			Yes
Area fixed effects: Planning area	Yes		
Area fixed effects: Subzone		Yes	Yes
Mean Dep Var.	24.1	24.0	23.7
Std. dev. of X	54.8	55.2	55.2
n(Transport facilities (area) within 1500m buffer > 0)	739	719	669
n(Clusters)	29	29	28
N	740	719	669

Note: Transport facilities area within 1500m buffer is the area (in 1,000 square meters) of the buffer area residence that is allocated to transport facilities land use. Figure A13 shows Model 2. Standard errors in (2)–(6) clustered at subzones. Significance levels: ^{c} 0.1 ^{b} 0.05 ^{a} 0.01.

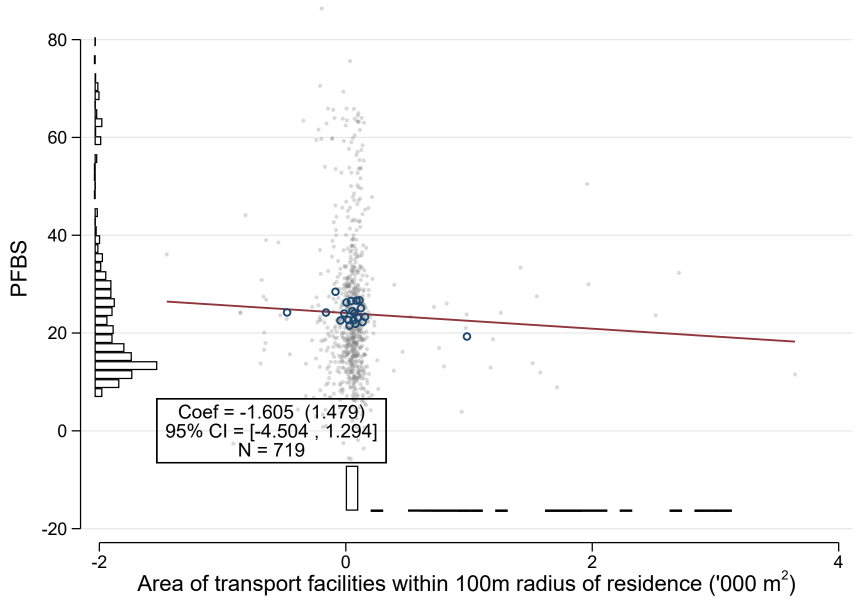

Figure A14. PFBS and Transport Facilities area within 100m radius. Reported estimates are from regressing PFBS on the area (1,000) of transport facilities within 1500m radius of residence, with subzone fixed effects and maternal characteristics partialed out. Model corresponds to column (2) of Table A7. Standard errors clustered at subzones. Raw data in small gray circles. Binned data in blue hollow circles. Fitted line from the estimated model. Marginal histograms indicate the underlying distributions. Significance levels: + 0.1 * 0.05 ** 0.01 *** 0.001.

Table A7. PFBS and transport facilities within 100m radius of residence

	Dependent variable is PFBS (ng/mL)		
	(1)	(2)	(3)
Transport facilities (area) within 100m buffer	-1.456	-1.605	-2.924^{c}
	(1.418)	(1.650)	(1.616)
	[-4.361–1.450]	[-4.986–1.775]	[-6.240–0.392]
	$< p = 0.313 >$	$< p = 0.339 >$	$< p = 0.082 >$
Constant	14.103	23.658	18.179
	(17.882)	(22.859)	(28.466)
	[-22.527–50.733]	[-23.167–70.482]	[-40.228–76.587]
	$< p = 0.437 >$	$< p = 0.310 >$	$< p = 0.528 >$
R^2	0.0944	0.191	0.216
Maternal baselines	Yes	Yes	Yes
Income			Yes
Area fixed effects: Planning area	Yes		
Area fixed effects: Subzone		Yes	Yes
Mean Dep Var.	24.1	24.0	23.7
Std. dev. of X	0.4	0.4	0.4
n(Transport facilities (area) within 100m buffer > 0)	34	33	30
n(Clusters)	29	29	28
N	740	719	669

Note: Transport facilities area within 100m buffer is the area (in 1,000 square meters) of the buffer area residence that is allocated to transport facilities land use. Figure A14 shows Model 2. Standard errors in (2)–(6) clustered at subzones. Significance levels: ^{c} 0.1 ^{b} 0.05 ^{a} 0.01.

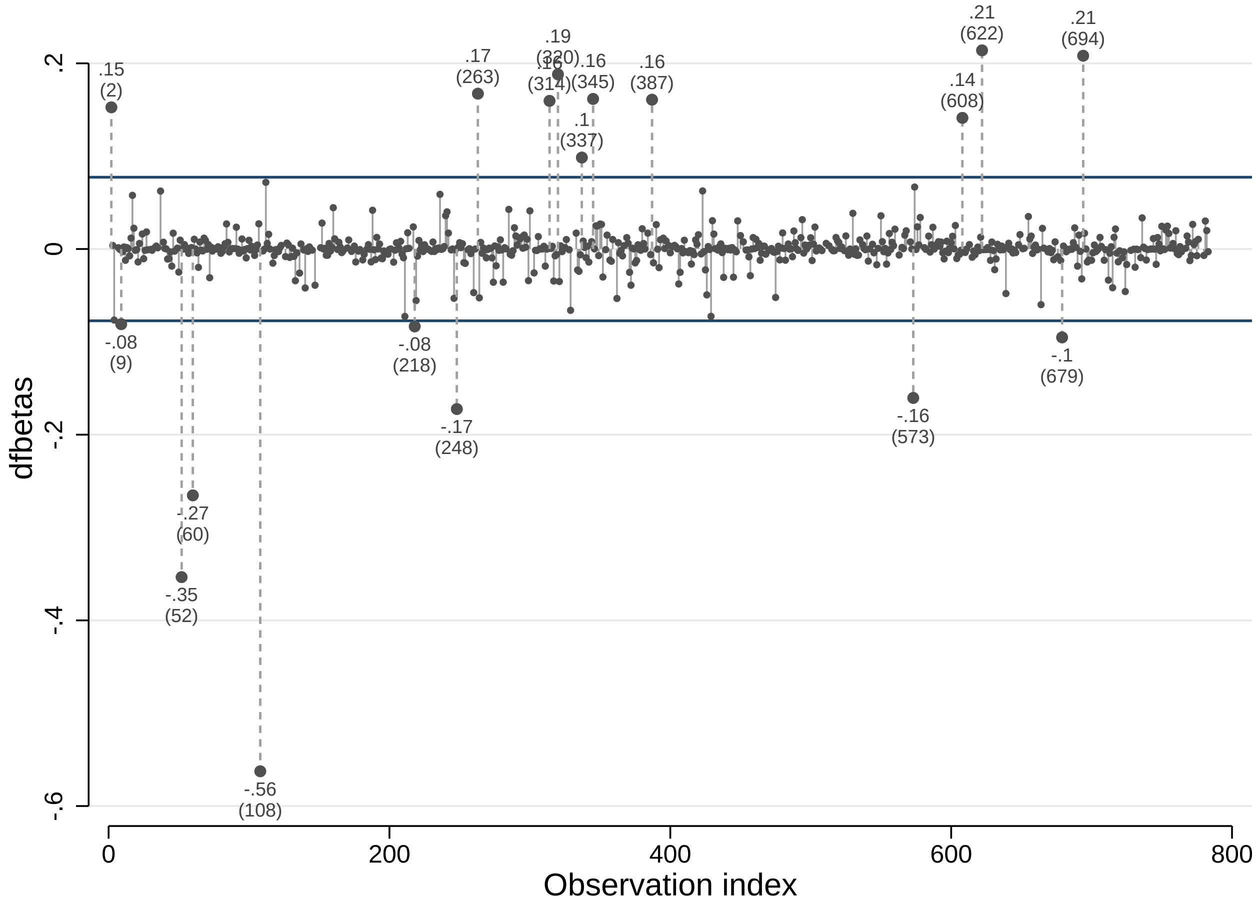

Figure A15. Figure shows the influence of each observation on the estimate of area of transport facilities within 500m radius of residence. Results corresponds to Figure A10 and Table A4. Vertical axis is the dfbeta measure standardized by the standard error of the estimate:

$$\text{dfbetas}_{(i)j} = \frac{\hat{\beta}_j - \hat{\beta}_{(i)j}}{SE(\hat{\beta}_j)}. \tag{A1}$$

where $\hat{\beta}_j$ is the estimate from omitting observation i. The paired horizontal lines are rule-of-thumb thresholds computed as $\pm 2/\sqrt{n}$. Observations falling outside the rule-of-thumb threshold highlighted with the dfbeta and observation index (in parentheses).

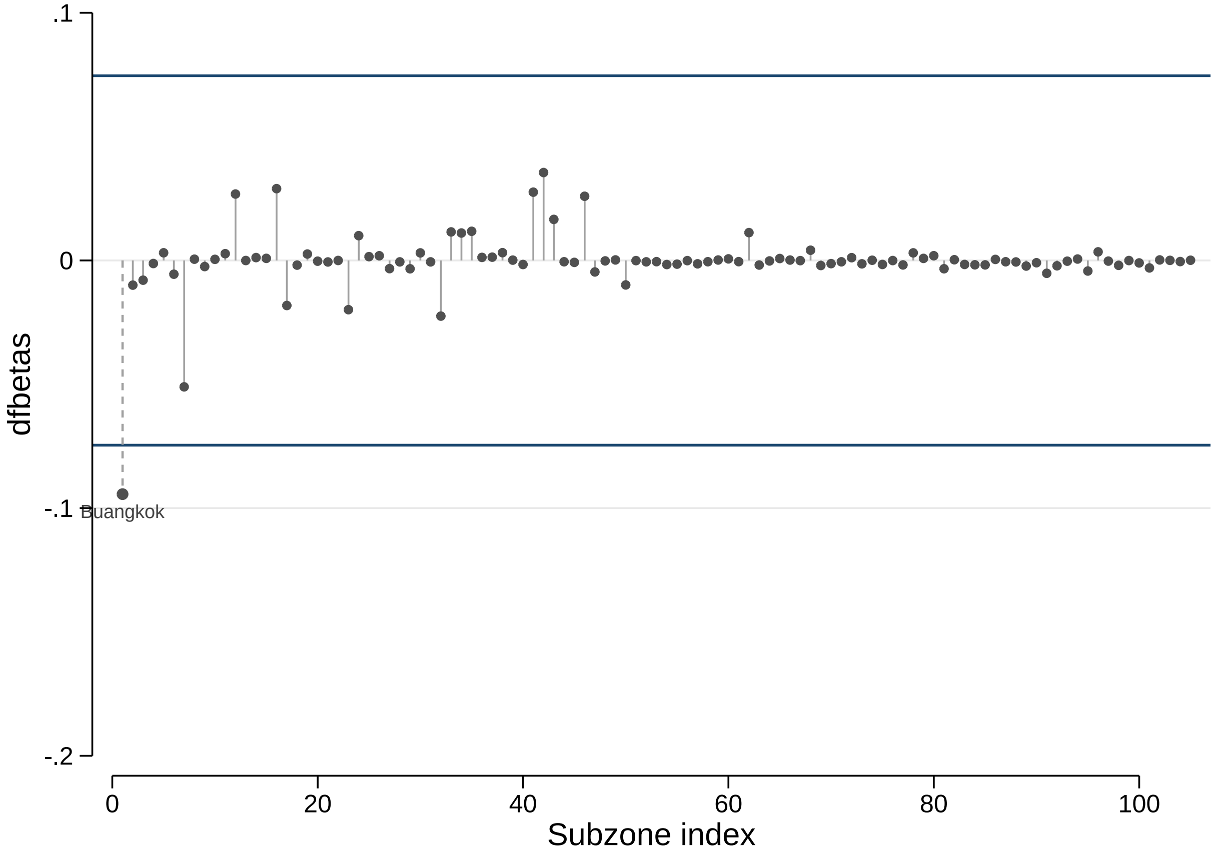

Figure A16. Figure shows the influence of each subzone on the estimations of Model 2 in Table A4. Vertical axis is the dfbetas measure standardized by the standard error of the estimate:

$$\text{dfbetas}_{(c)j} = \frac{\hat{\beta}_j - \hat{\beta}_{(c)j}}{SE(\hat{\beta}_j)}.$$

where $\hat{\beta}_c$ is the estimate from omitting all observations from subzone c. The paired horizontal lines are rule-of-thumb thresholds computed as $\pm 2/\sqrt{n}$. Subzones falling outside the rule-of-thumb threshold highlighted with the dfbeta and observation index (in parentheses).

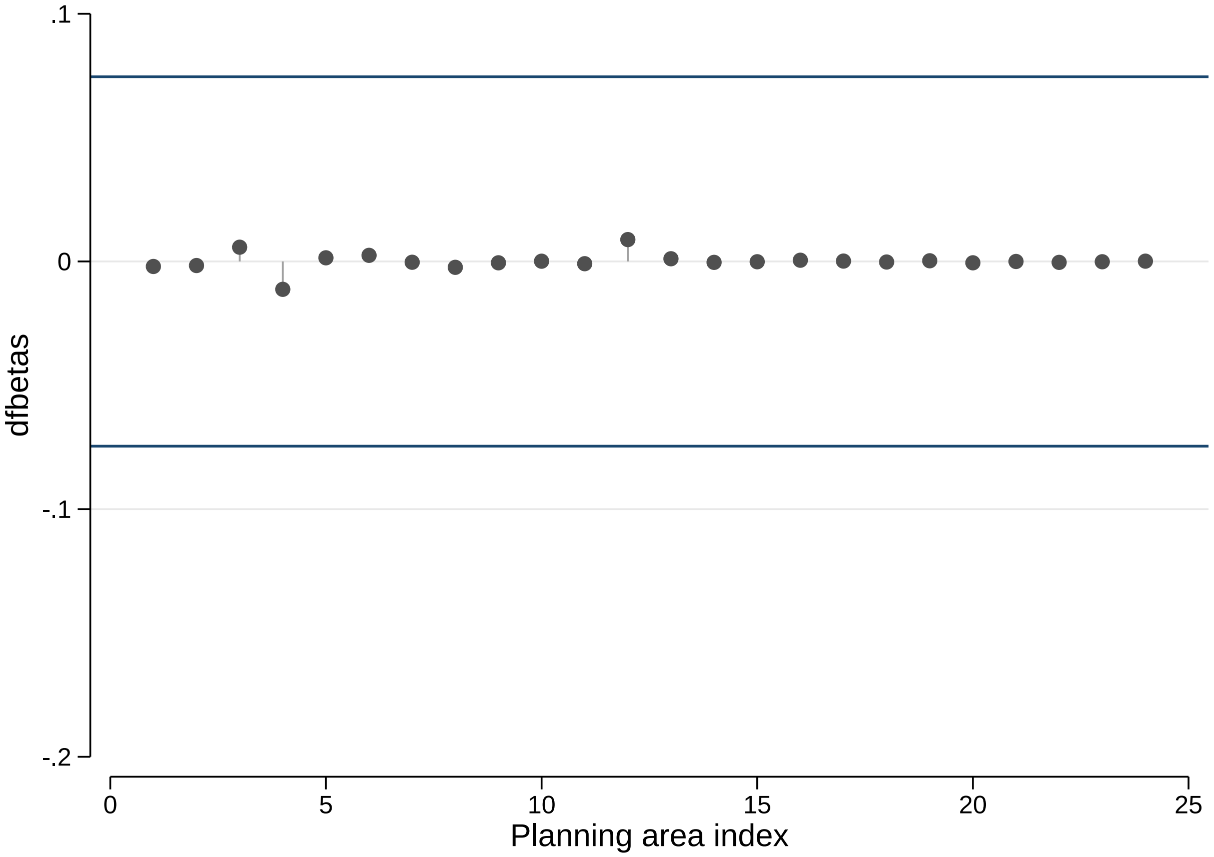

Figure A17. Figure shows the influence of each planning area on the estimations of Model 2 in Table A4. Vertical axis is the dfbetas measure standardized by the standard error of the estimate:

$$\text{dfbetas}_{(c)j} = \frac{\hat{\beta}_j - \hat{\beta}_{(c)j}}{SE(\hat{\beta}_j)}.$$

where $\hat{\beta}_c$ is the estimate from omitting all observations from planning area c. The paired horizontal lines are rule-of-thumb thresholds computed as $\pm 2/\sqrt{n}$. Planning areas falling outside the rule-of-thumb threshold highlighted with the dfbeta and observation index (in parentheses).

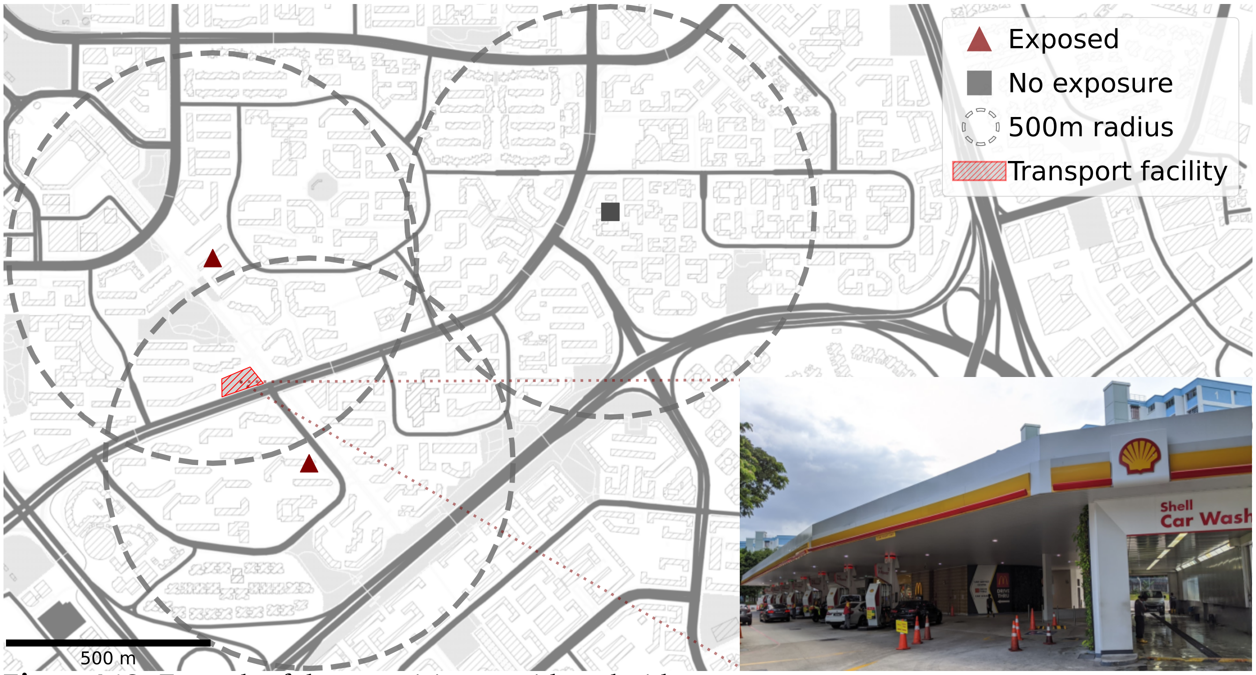

Figure A18. Example of three participants with and without exposure.
Image source: Google Photos.

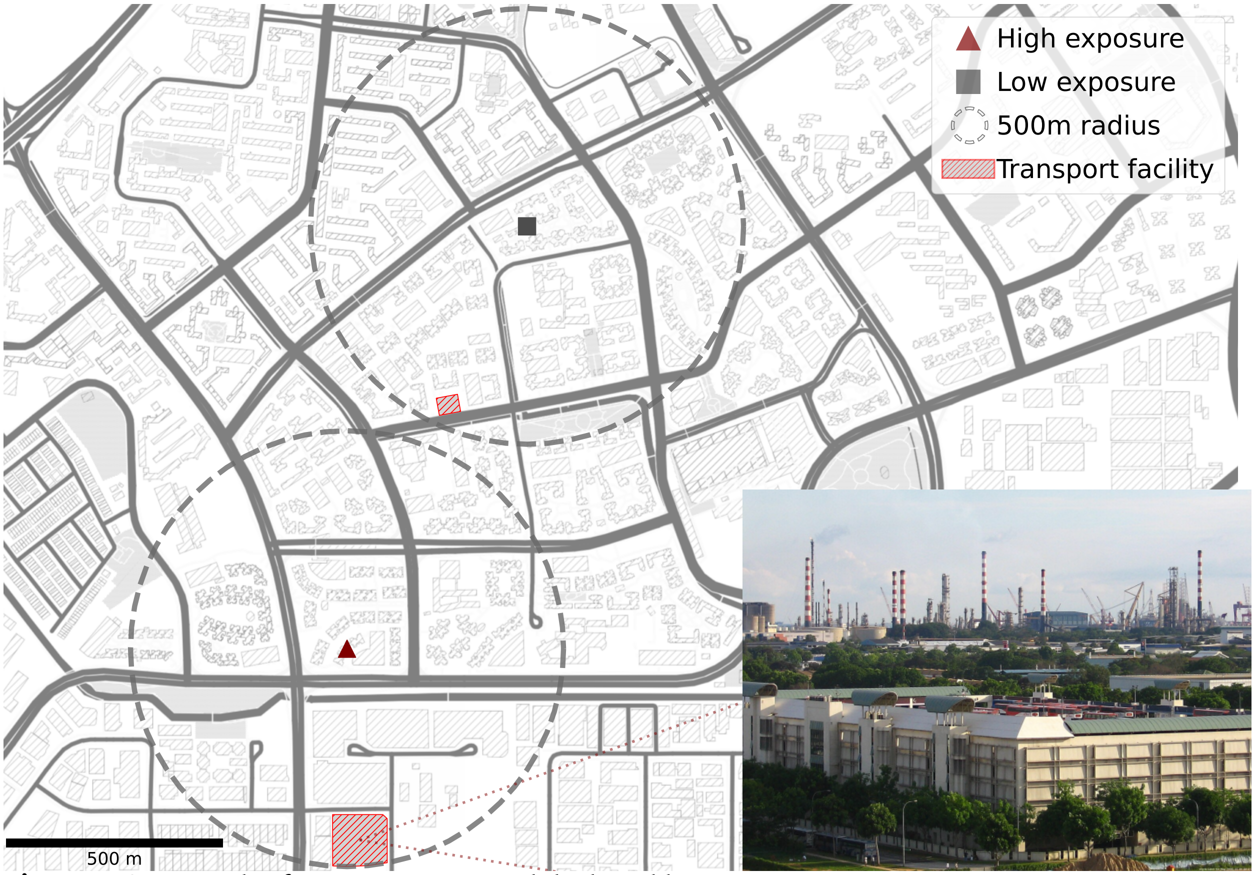

Figure A19. Example of two participants with high and low exposure.
Image source: https://en.wikipedia.org/wiki/Soon_Lee_Bus_Park#/media/File:Soon_Lee_Bus_Park_and_Jurong_Industrial_Estate_aerial_view.jpg.

B. Public-contributed street-level pictures of transport facilities

This appendix provides further details about how we attempted to pin down the specific type of transport facility using the publicly available land use data. We know where land parcels for transport facilities are located, but not the specific facility type.

To map residences to land use zones of potential hazard in our main analyses, we use the publicly-available vector data on land zones with assigned typology, such as Transport Facility. Within each land use type, there can be further breakdowns of types. For instance, a transport facility can be one of eight subtypes: 1) car park, 2) heavy vehicle park, 3) trailer park, 4) bus depot/terminal, 5) transport depot, 6) MRT/LRT marshalling yard/depot, 7) driving circuit/test centre, or 8) petrol station/kiosk (Table A3). This specificity on the subtypes, however, is not present in the public data. For instance, for a given parcel of land zone assigned for transport facilities, we do not know if this would be a gas station or a transit depot.

To address this and to partially scale the solution, we systematically obtain street-level public-contributed images geotagged to the location of the transport facilities. There are, in total, 363 land parcels assigned to transport facilities in the 2008 data. We first reverse-geocode all the 298 transport facilities whose centroids are within 1000m of a GUSTO residence, using the centroid coordinate.[1] From this, we obtain the associated formatted address, Google Place ID, and Google Place type. We successfully recovered these for 246 transport facility land parcels. The place types for these 246 are tabulated in Table B8.

To further gain insights into the transport facility land use, we use the 246 place ID to obtain a list of photo IDs tagged to the place,[2] before finally using the photo IDs to download the photos.[3] We successfully obtained at least one street-level photo for 193 of the transport facility land parcels. In total, we have 1135 photos, and we looked through all of them. Figure B20 shows an assort of photos related to gas stations while Figure B23 shows an assortment of photos broadly related to transit depots. The Places service also returns the name of the point of interest, if available, but we do not find this to be a particularly clear signal of transport facility type. For instance, one place name we recovered is "LKA Auto Parts Trading Pte Ltd," but upon inspection of the tagged photos, it became apparent that it is a gas station (image D in Figure B20).

Each place can have more than one type. The "Establishment" and "Point of Interest" type gets tagged to most ($n = 208$). Store, food, and convenience stall also get tagged to many transport facility ($n \geq 58$). This does not rule out our queried land parcels as

[1] Using the Google Geocoding Service: https://developers.google.com/maps/documentation/javascript/geocoding.

[2] Using the Google Places Service: https://developers.google.com/maps/documentation/places/web-service/details.

[3] Using the Google Photos Service: https://developers.google.com/maps/documentation/places/web-service/photos.

transport facilities. Virtually all gas stations/petrol kiosks in Singapore have convenience shops that also sell food (Figure B20 and Figure B21). Many of the tagged pictures we later see (Figure B21) are also of these known convenience stalls (e.g., 7-Eleven, FairPrice Xpress, etc.) commonly found in gas stations, more on the pictures below. Other observations are that 58 are typed as gas stations, 20 as car wash (which can also be found in gas stations, Figure B22), 17 as transit stations, 2 as subway station, and 1 as a bus station.

Overall, many photos of transport facilities appear to be gas stations/petrol kiosks or transit depots. The assumption here is that the other types of transport facilities (Table A3) are rare and do not have some attribute that makes them less likely to be captured in the places and photos database or less likely to be captured in pictures by the public. Linking the photos to the underlying spatial data, we observe that gas stations occupy relatively small land areas. In Figure B20, the gas station in subfigure (a) has a known land area (and closest centroid-to-point distance to a GUSTO residence point) of 1,190m^2 (414m), the gas station in subfigure (b) is 800m^2 (180m), the gas station in subfigure (b) is 1,862m^2 (898m), and the gas station in subfigure (d) is 2,600m^2 (214m). The land parcels that appear to be transit depots are far larger. The bus depot in subfigure (a) of Figure B23 is 52,608m^2, and the closest centroid-to-point (not edge-to-point) distance to a GUSTO residence point is 963m. The bus depot in Figure 1 is 25,981m^2 with the nearest GUSTO residence within 592m. For reference, one of the larger gas stations we find is 4,055m^2 and is within 976m of a GUSTO residence (Figure B24). All street-level photos (Figures B20 to B24) in this manuscript are public contributed but taken directed via the Google Photos API.

Table B8. Types of detected places in transport facilities parcels

	Type	Count
1	Establishment	208
2	Point Of Interest	208
3	Store	80
4	Food	67
5	Convenience Store	58
6	Gas Station	58
7	Car Wash	20
8	Atm	19
9	Finance	19
10	Transit Station	17
11	Car Repair	14
12	Restaurant	13
13	Grocery Or Supermarket	6
14	Supermarket	6
15	Place Of Worship	4
16	Park	3
17	Meal Delivery	3
18	Parking	3
19	Subway Station	2
20	Cafe	2
21	Bakery	2
22	Clothing Store	1
23	Tourist Attraction	1
24	Street Address	1
25	Health	1
26	General Contractor	1
27	Bus Station	1
28	Car Rental	1
29	Home Goods Store	1
30	Car Dealer	1

Note: Types come from the Google Geocoding API. See Figure 2. For n = 246 Google places. Types are not mutually exclusive, places can have multiple types. Types are not necessarily accurate nor complete.

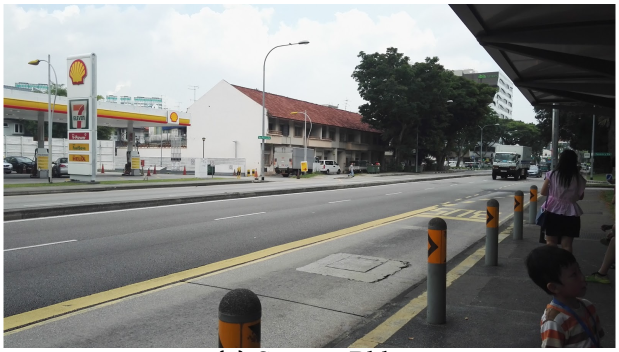

(a) Cencon Bldg

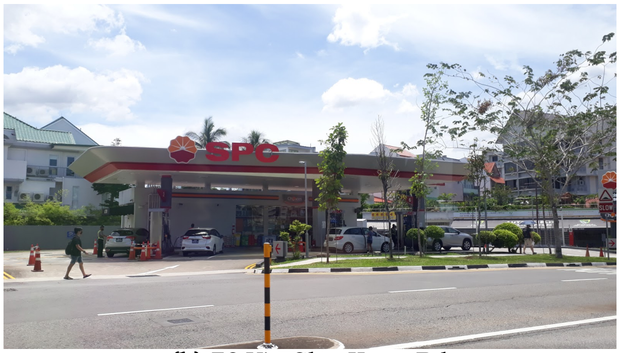

(b) 76 Yio Chu Kang Rd

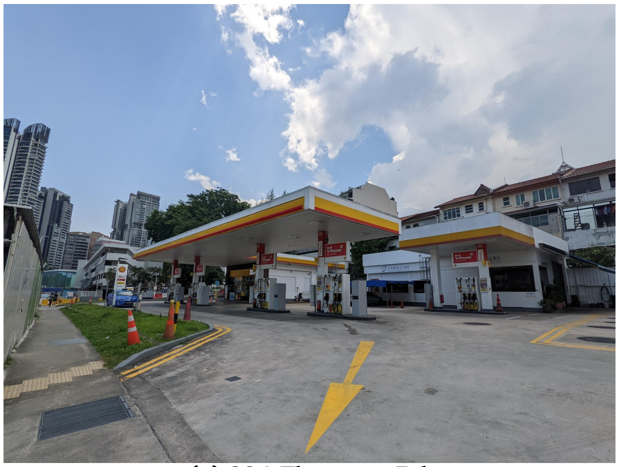

(c) 324 Thomson Rd

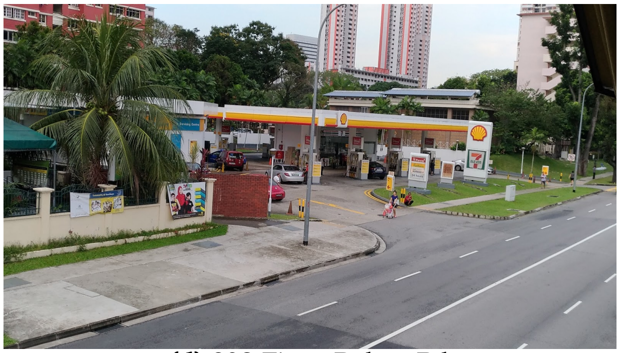

(d) 603 Tiong Bahru Rd

Figure B20. Petrol Stations. Selected illustrations of petrol stations and kiosks in transport facilities land parcels within 1000m of known (GUSTO) residences. Photos are from the Google Place Photo service which source from photos that are user-submitted and photos from managers of establishments.
Image source: Google Photos.

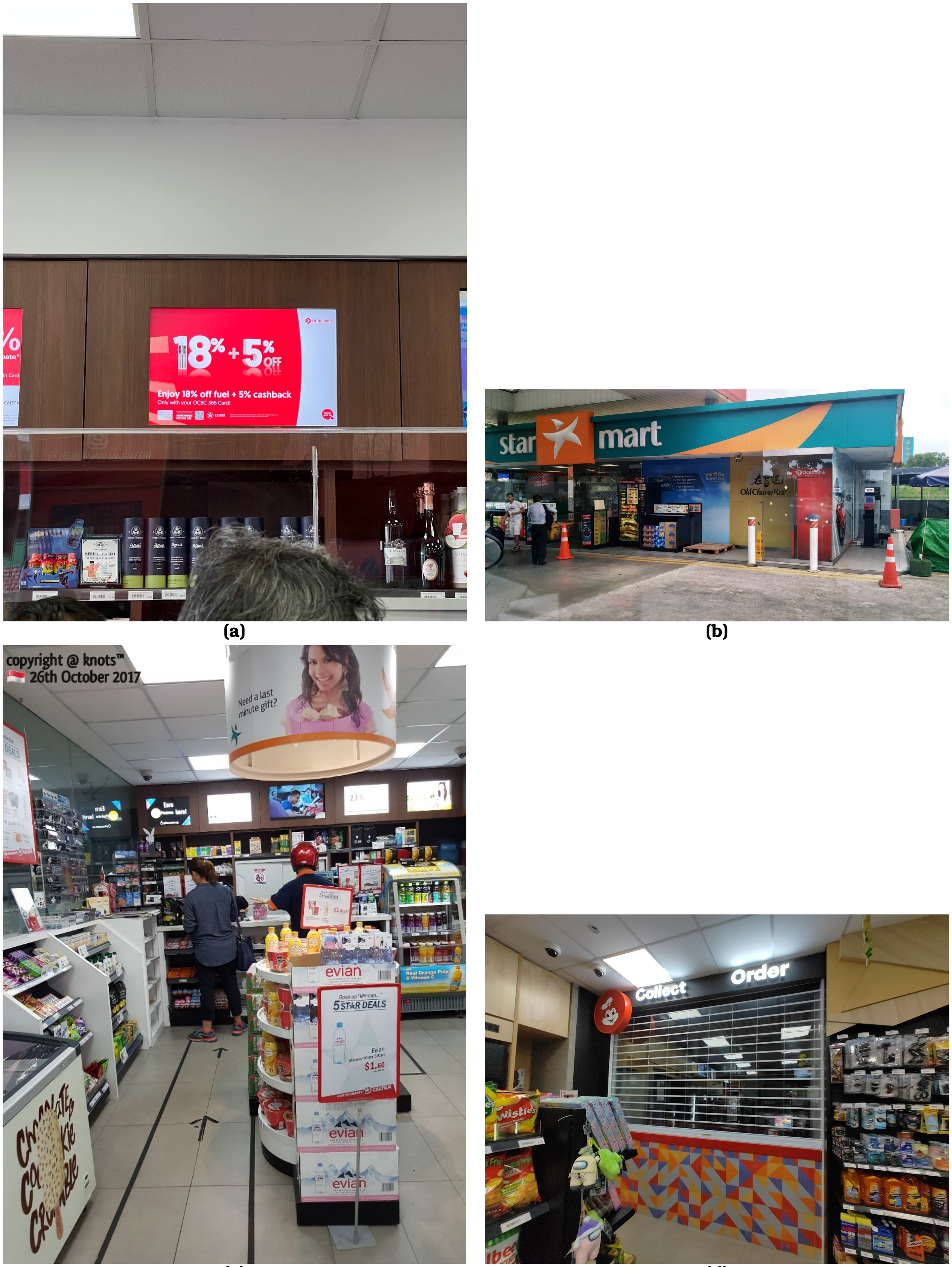

(a) (b) (c) (d)

Figure B21. Food and convenience shops in gas stations.
Image source: Google Photos.

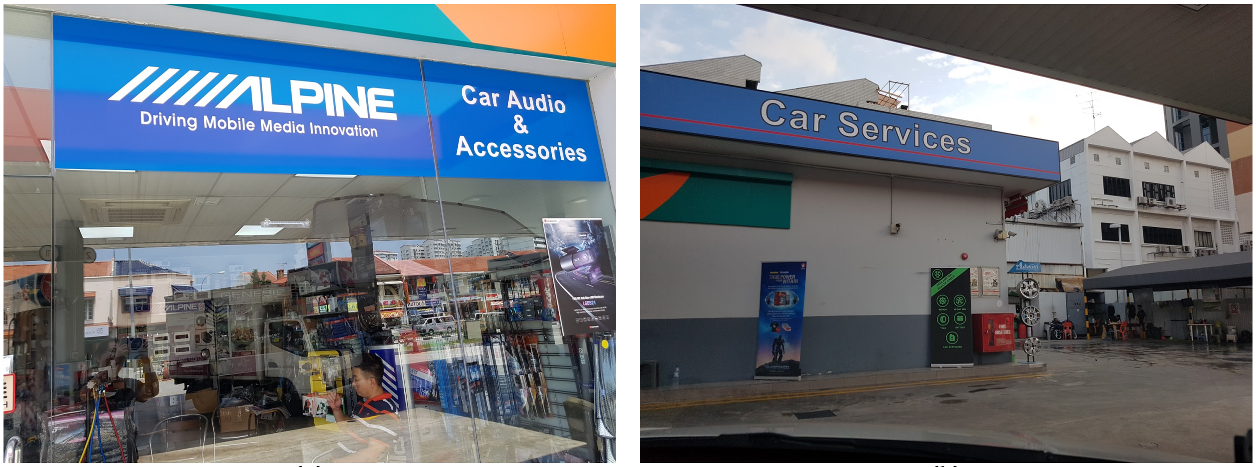

(a) (b)

Figure B22. Car wash and car repair services in gas stations.
Image source: Google Photos.

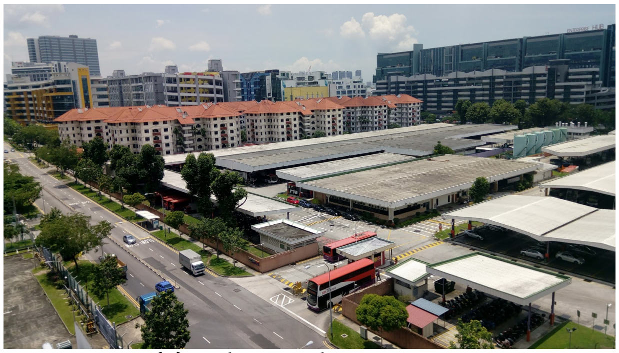

(a) Bukit Batok Bus Depot

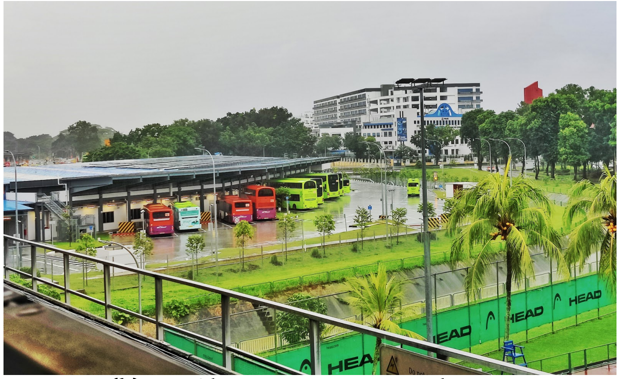

(b) Yio Chu Kang Bus Interchange

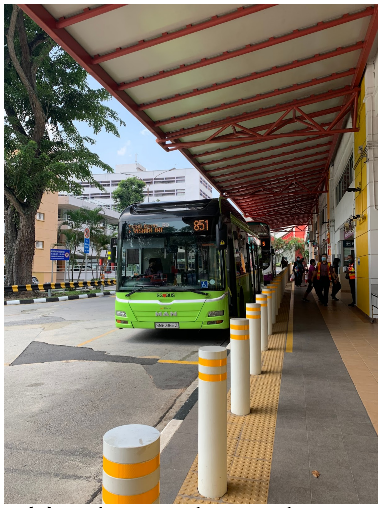

(c) Bukit Merah Interchange

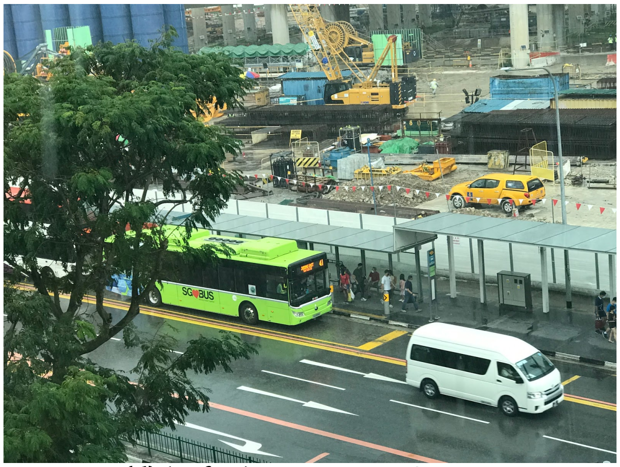

(d) (Before) Jurong East Station

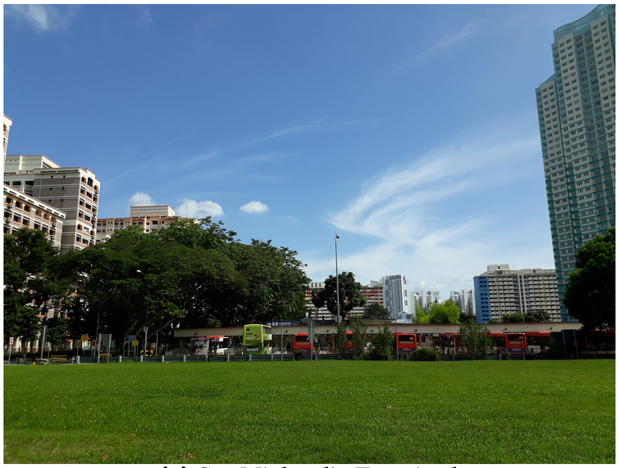

(e) St. Michael's Terminal

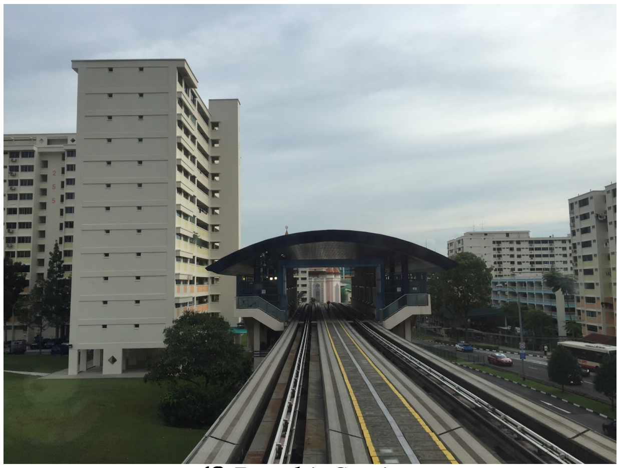

(f) Bangkit Station

Figure B23. Transport depots, terminals, and stations. Selected illustrations of transport depots in transport facilities land parcels within 1000m of known (GUSTO) residences. Photos are from the Google Place Photo service which source from photos that are user-submitted and photos from managers of establishments.
Image source: Google Photos.

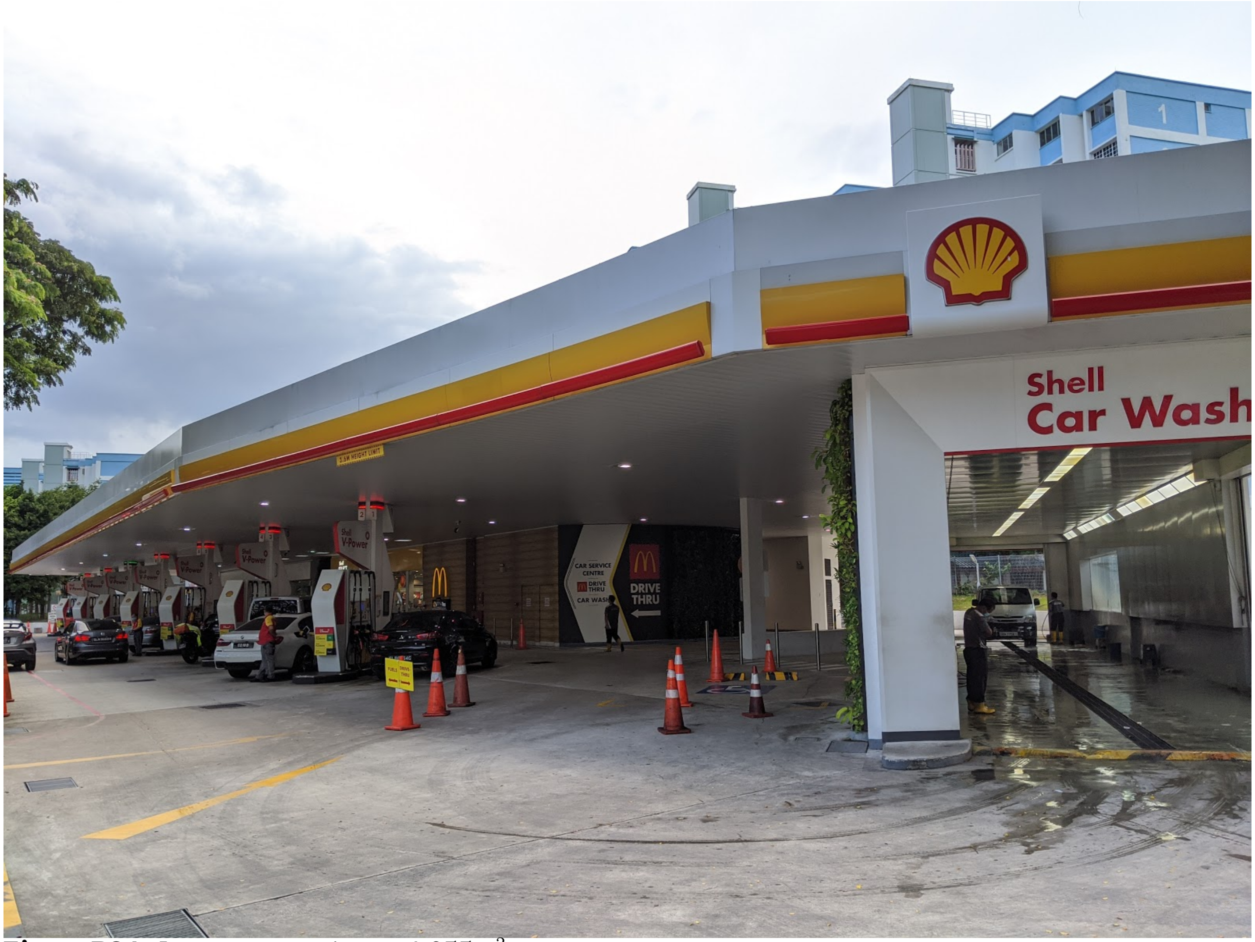

Figure B24. Larger gas station at 4,055m^2.
Image source: Google Photos.

C. Other land uses

This appendix considers and reports results for all other land use types. The spatial vector data on land parcels and their land uses come from the URA official land use data in Singapore. For the main evaluations, we use the 2008 version, which is closest to when blood samples were collected, where there are more than 110k delineated land parcels of 32 land types over 776km^2 of land in the 2008 plan.

Figure C25 reports the within-subzone correlation of transport facilities with all other land use types. The measures of the land uses are as defined in Section 2.3 of the main article and share the same units. We observe that four land use types—Place of Worship, Residential, Business 1 - White, and Port/Airport—are negatively correlated with the transport facility land use. Another four land use types—Residential/Institution, Business 2, Business Park - White, and Business 2 - White—are positively correlated with the transport facility land use. Business 1 and Business 2 broadly refers to light and heavy industry uses, respectively (see https://web.archive.org/web/20240717071728/https://www.ura.gov.sg/-/media/Corporate/Planning/Master-Plan/MP19writtenstatement.pdf?la=en#page=11.) The estimated coefficients are adjusted for subzone areal effects and standard errors are clustered at the planning areas.

Figure C26 reports the estimated association between the plasma PFBS concentration with all the other land uses, in addition to transport facilities. We observe statistically significant and fairly large estimated coefficients for Business 2 - White and Light Rapid Transit (LRT). Although it may be interpreted as a signal of potentially hazardous sites, we also note that in 2008, there were only three Business 2 - White land parcels (14 today) with a collective land area of 0.09km^2 (0.17km^2 today). For the LRT, in 2008 there were only 14 parcels (15 today) with a collective land area of 0.01km^2 (0.02km^2 today).

General industrial areas. Factories and industrial plants are the known land parcels of concern. However, residences near industrial sites in our urban setting are rare. General industrial ("Business 2") areas have a regulatory minimum nuisance buffer of 100m (approximately 330 feet). In 2008, there were 5,576 land parcels used for heavy industry with a collective land area of 118km^2 (approximately 15.2% of total land area), but most are geographically clustered outside residential neighborhoods (Figure A1). In practice, the industrial areas also usually more than clear the regulatory minimum. The nearest distance from our participants to a general industrial land use parcel is 45 meters by edge of the land parcel 70 meters by of land parcel, and only 163 participants have an industrial land use parcel intersecting within 500m of their residence. For that single residence within 45 meters of a industrial land parcel, we checked and the corresponding industrial site is small on average—with a land parcel of approximately 2,000m^2—which is small relatively to the collectively areas of industrial sites on the outskirts of neighborhoods (see Figure A1). Using distance to centroid, only one residence is within 100 meters. For distance to edge, 18 residences are within 100 meters.

Transport facilities do not share these regulatory buffers and can locate right next to residential buildings (Figure A18 and Figures A2 to A7). For distance to centroid, 33 residences are within 100 meters of a transport facility. For distance to edge, 72 residences are within 100 meters of a transport facility. By distance to edge (distance to centroid), residences in our sample with blood collections live as near as within 18 meters (40 meters) of a transport facility. There are 363 transport facility land parcels with a collective land area of 2.3km^2 (approximately 0.3% of total land area). Base on distance to edge (distance to centroid), the nearest transport facility is within 18 meters (40 meters) of a residence. While the collective land area is fairly small, these transport facilities are scattered geographically. For additional context, we use about 120k of known postal codes and compute the percentage of area attributed transport facilities and industrial land parcels within a 500m concentric circle as 0.38% (SD 1.1%) and 4.83% (SD 15.79%), respectively. When we subset postal codes to known public residences, the numbers change to 0.41% (SD 1.27%) and 1.63% (SD 1.27), suggesting that the industrial areas tend to be placed further away from residential areas.

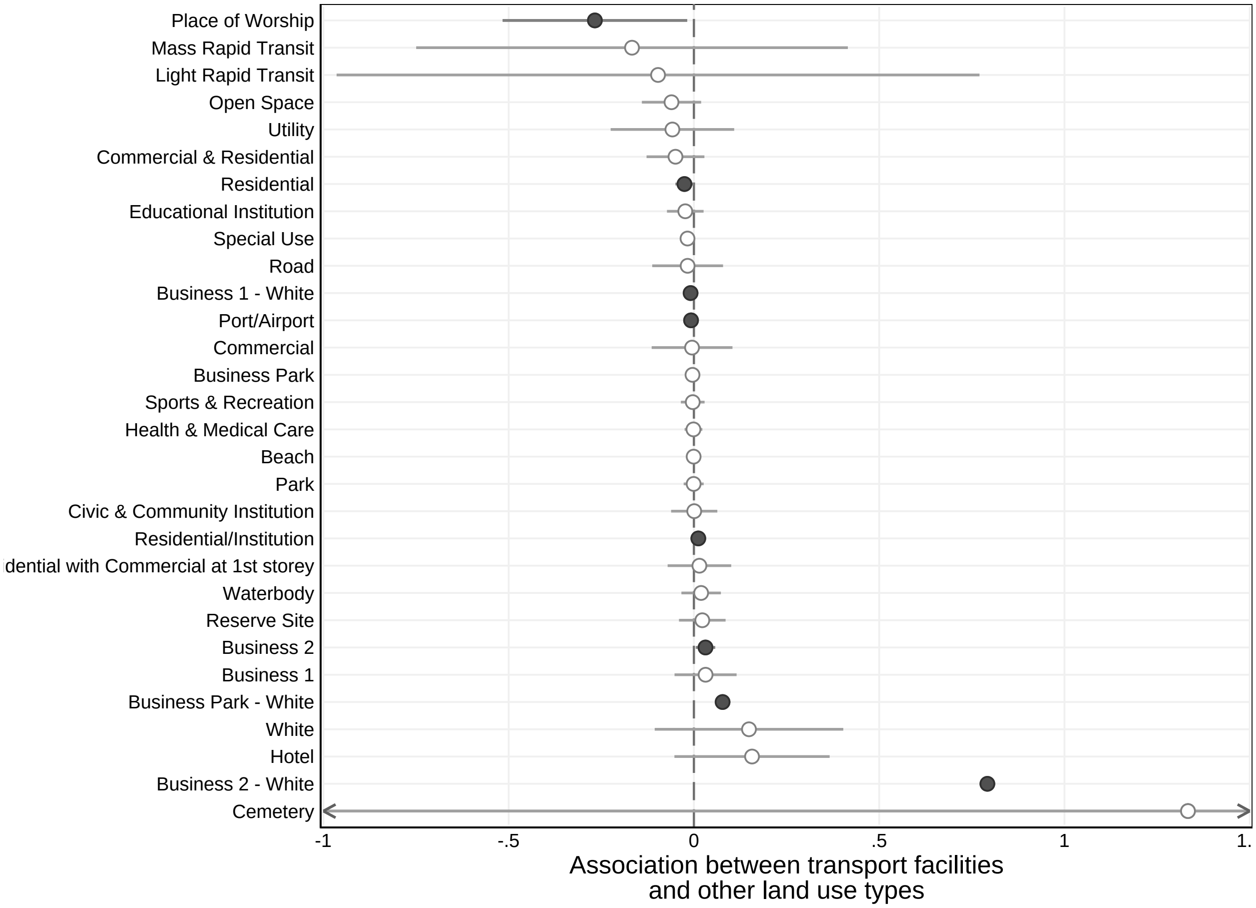

Figure C25. Correlation of transport facilities to other land use types. Horizontal axis reports the $\hat{\beta}$ coefficients from estimating

$$\text{transport facilities}_{ic} = \beta(\text{land use})^{\text{j}}_{ic} + \delta_c(\text{subzone})_c + \varepsilon_{ic}, \tag{C2}$$

where $(\text{land use})^{\text{j}}$ is one of the land use types indicated on the vertical axis. All variables are area within 500m radius of residence. Estimates significant at the 5% level have black markers; the remaining estimates have hollow markers. Gray horizontal lines are the 95% confidence intervals constructed from standard errors clustered at planning areas. Arrows indicate truncated confidence intervals.

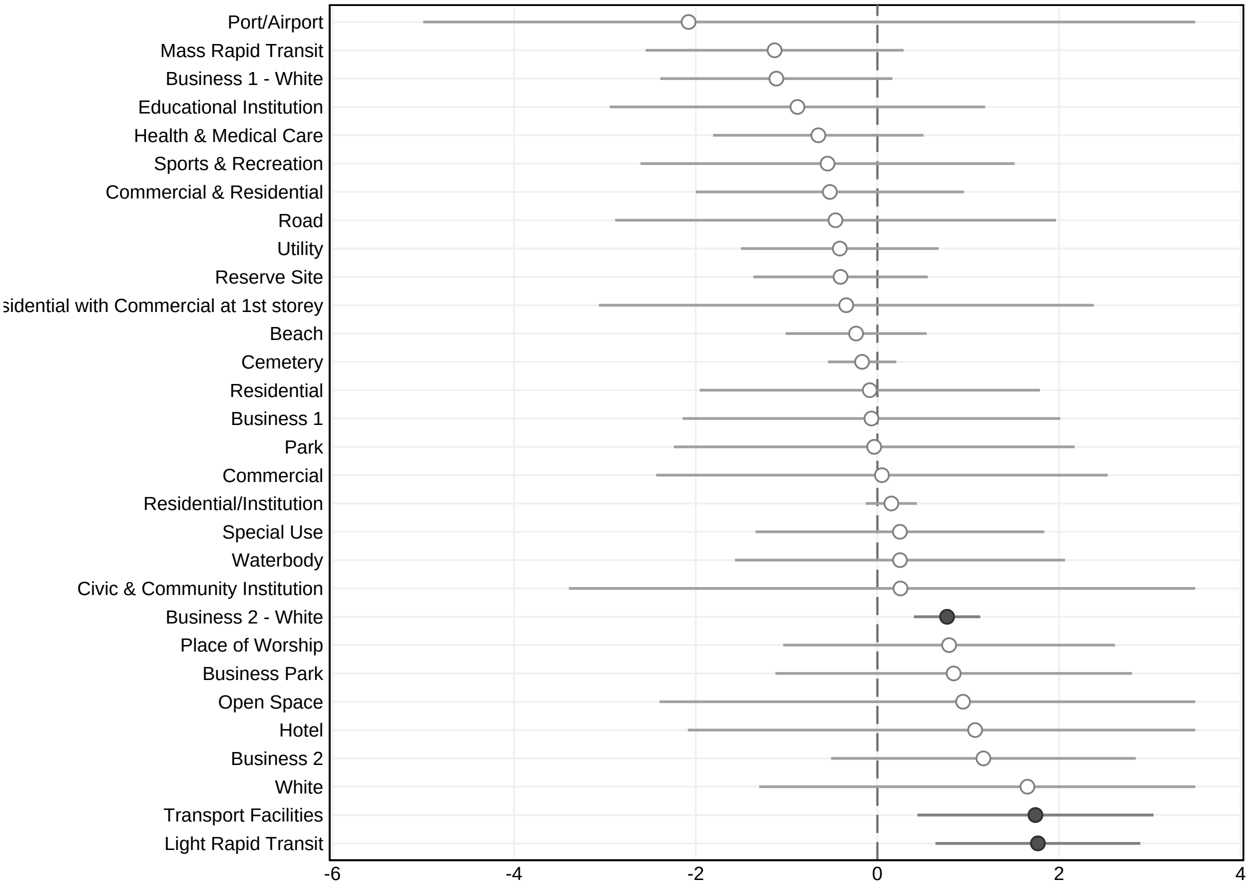

Figure C26. Association between PFBS and land use types. Horizontal axis reports the $\hat{\beta}$ coefficients from estimating

$$\text{PFBS}_{ic} = \beta(\text{land use})^{j}_{ic} + \gamma X_i + \delta_c(\text{subzone})_c + \varepsilon_{ic}, \tag{C3}$$

where (land use)^{j} is area (units in 1'000m^2) of the land use type indicated on the vertical axis. Model is equivalent to model (5) of Table A4. Estimates significant at the 5% level have black markers. Gray horizontal lines are the 95% confidence intervals constructed from standard errors clustered at planning areas. Arrows indicate truncated confidence intervals.

D. Other spatial instruments

To help examine nuanced spatial patterns in the areal proximity to transport facilities, and as an auxiliary exploration of the exposure mechanism, this appendix evaluates a set of alternative spatial instruments of the exposure measure defined in Section 2.3.

The four alternative instruments are: i) distance to nearest transport facility by a point-to-edge distance from the point of residence to the edge of the land parcel, ii) distance to nearest transport facility by a point-to-centroid distance from the point of residence to the geometric centroid of the land parcel, iii) number of transport facilities within the concentric circle, and iv) accessibility-based area around residence based on areas within 10–20-minute reach by public transit (including walking). As a caveat, the public travel time data was collected around 2021. These alternative spatial instruments that are potentially linked to the main exposure measure defined in Section 2.3 through spatial patterns relating to where residences and transport facilities according to urban planning, but potentially have no direct pathway to PFAS exposure. More concretely, if exposure is also tied to the size of the facility, residences near transport facilities are likely similar to residences with high exposure as defined in Section 2.3 but otherwise should not share the same level of exposure. Figure D27 reports the correlation between the four key alternative instruments (p-values $< .001$).

Table D9 report the estimates for the different models for the point-to-edge distance to the nearest transport facility. Models and adjustments are otherwise similar to Equation (1) and Table A4. We observe no association between proximity measured by distance to nearest transport facility and the plasma PFBS concentration. From Model 2, the estimated coefficient is -0.676 (SE 3.38, p = .843, Table D9), which implies that a standard deviation decrease in distance to the nearest transport facility ($\sim$300m) is related to a -0.2 decrease in PFBS (SE 0.01). Table D10 reports the estimates for nearest distance to transport facilities measured as point of residence to the geometric centroid of the land parcel. We do not observe any statistically significant association here.

Table D11 reports the estimates using the number instead of total area of transport facilities in the concentric circle of 500m radius. The estimated coefficients here are consistently positive but not statistically significant at conventional levels. In Model 2, the estimated coefficient is 0.911 (SE 0.614, p = .149, Table D11), which implies that an additional land parcel for transport facility within the concentric circle is associated with a 0.9 point increase in the plasma PFBS concentration (0.06 of the SD).

Table D13 reports the estimation using a 15-min travel buffer around residences to compute exposure. The estimated coefficients across the models are negative but not statistically significant. The estimated association for Model 2 is -0.014 (SE 0.069, p = .841, Table D13). Using a 10-min buffer reported in Table D12 and a 20-min buffer reported in Table D14 yield similar null findings.

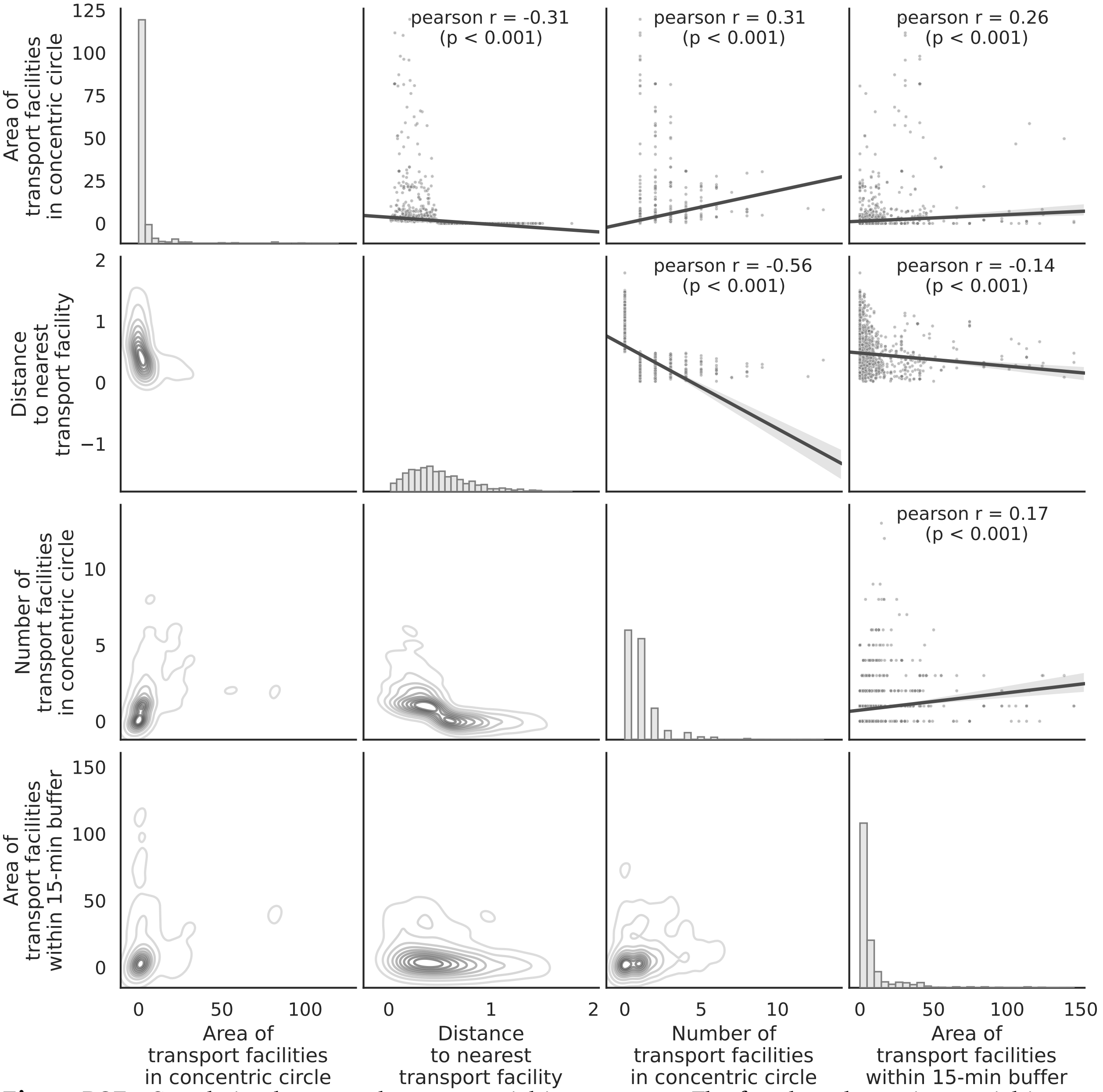

Figure D27. Correlation between alternate spatial instruments. The four key alternative spatial instruments of transport facilities are: (1) area of transport facilities within concentric cirlce (main instrument in Section 2.3), (2) distance to nearest transport facilities, (3) number of transport facilities in concentric circle, and (4) area of transport facilities within 15-minute of residence. Confidence intervals are bootstrapped (n = 1000). Main diagonal reports the univariate histogram.

Table D9. PFBS and (point-to-edge) distance to nearest transport facility

	Dependent variable is PFBS (ng/mL)		
	(1)	(2)	(3)
Distance to nearest transport facility's edge	−0.001	−0.001	0.001
	(0.002)	(0.003)	(0.003)
	[−0.005–0.003]	[−0.008–0.006]	[−0.006–0.008]
	$< p = 0.524 >$	$< p = 0.854 >$	$< p = 0.712 >$
Constant	15.882	25.272	19.291
	(18.849)	(24.877)	(30.412)
	[−22.729–54.493]	[−25.686–76.230]	[−43.110–81.691]
	$< p = 0.407 >$	$< p = 0.318 >$	$< p = 0.531 >$
R^2	0.0938	0.190	0.212
Maternal baselines	Yes	Yes	Yes
Income			Yes
Area fixed effects: Planning area	Yes		
Area fixed effects: Subzone		Yes	Yes
Mean Dep Var.	24.1	24.0	23.7
Std. dev. of X	298.3	293.5	293.1
n(Distance to nearest transport facility's edge > 0)	740	719	669
n(Clusters)	29	29	28
N	740	719	669

Note: The main independent variable—Distance to nearest transport facility—is the nearest distance from the given participant residence to the nearest transport facility land parcel computed as point-to-edge distance. Other than the spatial instrument of exposure to transport facilities, models and adjustments are the same as in Equation (1) and Table A4. Standard errors in (2)–(6) clustered at subzones. Significance levels: ^{c} 0.1 ^{b} 0.05 ^{a} 0.01.

Table D10. PFBS and (point-to-centroid) distance to nearest transport facility

	Dependent variable is PFBS (ng/mL)		
	(1)	(2)	(3)
Distance to nearest transport facility's centroid	−0.001	−0.001	0.001
	(0.002)	(0.003)	(0.003)
	[−0.005–0.003]	[−0.008–0.006]	[−0.006–0.008]
	$< p = 0.524 >$	$< p = 0.854 >$	$< p = 0.712 >$
Constant	15.882	25.272	19.291
	(18.849)	(24.877)	(30.412)
	[−22.729–54.493]	[−25.686–76.230]	[−43.110–81.691]
	$< p = 0.407 >$	$< p = 0.318 >$	$< p = 0.531 >$
R^2	0.0938	0.190	0.212
Maternal baselines	Yes	Yes	Yes
Income			Yes
Area fixed effects: Planning area	Yes		
Area fixed effects: Subzone		Yes	Yes
Mean Dep Var.	24.1	24.0	23.7
Std. dev. of X	298.3	293.5	293.1
n(Distance to nearest transport facility's centroid > 0)	740	719	669
n(Clusters)	29	29	28
N	740	719	669

Note: The main independent variable—Distance to nearest transport facility—is the nearest distance from the given participant residence to the nearest transport facility land parcel computed as point-to-centroid distance in meters. Other than the spatial instrument of exposure to transport facilities, models and adjustments are the same as in Equation (1) and Table A4. Standard errors in (2)–(6) clustered at subzones. Significance levels: ^{c} 0.1 ^{b} 0.05 ^{a} 0.01.

Table D11. PFBS and the number of transport facilities within 500m

	Dependent variable is PFBS (ng/mL)		
	(1)	(2)	(3)
Transport facilities within 500m	0.831	0.924	0.394
	(0.498)	(0.635)	(0.687)
	[−0.190–1.852]	[−0.376–2.223]	[−1.015–1.802]
	$< p = 0.107 >$	$< p = 0.157 >$	$< p = 0.571 >$
Constant	14.469	24.881	20.529
	(18.472)	(23.557)	(29.181)
	[−23.370–52.307]	[−23.373–73.135]	[−39.347–80.404]
	$< p = 0.440 >$	$< p = 0.300 >$	$< p = 0.488 >$
R^2	0.0976	0.193	0.213
Maternal baselines	Yes	Yes	Yes
Income			Yes
Area fixed effects: Planning area	Yes		
Area fixed effects: Subzone		Yes	Yes
Mean Dep Var.	24.1	24.0	23.7
Std. dev. of X	1.4	1.4	1.4
n(Transport facilities within 500m > 0)	438	425	398
n(Clusters)	29	29	28
N	740	719	669

Note: The main independent variable is the number of transport facilities within the 500m concentric circle. Models and adjustments are otherwise the same as in Equation (1) and Table A4. Standard errors in (2)–(6) clustered at subzones. Significance levels: ^{c} 0.1 ^{b} 0.05 ^{a} 0.01.

Table D12. PFBS and transport facilities within 10 min of residence

	Dependent variable is PFBS (ng/mL)		
	(1)	(2)	(3)
Transport facilities (area) within 10min	−0.106	−0.161	−0.153
	(0.216)	(0.249)	(0.231)
	[−0.550–0.339]	[−0.673–0.351]	[−0.628–0.321]
	$< p = 0.630 >$	$< p = 0.525 >$	$< p = 0.513 >$
Constant	13.345	24.472	19.515
	(18.132)	(23.364)	(28.035)
	[−23.858–50.548]	[−23.468–72.411]	[−38.112–77.142]
	$< p = 0.468 >$	$< p = 0.304 >$	$< p = 0.493 >$
R^2	0.0885	0.190	0.215
Maternal baselines	Yes	Yes	Yes
Income			Yes
Area fixed effects: Planning area	Yes		
Area fixed effects: Subzone		Yes	Yes
Mean Dep Var.	24.1	23.9	23.7
Std. dev. of X	2.9	3.0	3.0
n(Transport facilities (area) within 10min > 0)	279	269	246
n(Clusters)	28	28	27
N	714	694	645

Note: Transport facilities area is the area (in 1,000 square meters) within 15 minutes of residence that is allocated to transport facilities land use. Models and adjustments are otherwise the same as in Equation (1) and Table A4. Standard errors in (2)–(6) clustered at subzones. Significance levels: ^{c} 0.1 ^{b} 0.05 ^{a} 0.01.

Table D13. PFBS and transport facilities within 15 min of residence

	Dependent variable is PFBS (ng/mL)		
	(1)	(2)	(3)
Transport facilities (area) within 15min	0.039	0.021	0.040
	(0.058)	(0.082)	(0.077)
	[−0.081–0.158]	[−0.147–0.189]	[−0.117–0.198]
	$< p = 0.509 >$	$< p = 0.802 >$	$< p = 0.602 >$
Constant	12.934	24.307	18.876
	(18.281)	(23.480)	(28.132)
	[−24.576–50.443]	[−23.870–72.485]	[−38.951–76.702]
	$< p = 0.485 >$	$< p = 0.310 >$	$< p = 0.508 >$
R^2	0.0890	0.189	0.215
Maternal baselines	Yes	Yes	Yes
Income			Yes
Area fixed effects: Planning area	Yes		
Area fixed effects: Subzone		Yes	Yes
Mean Dep Var.	24.1	23.9	23.7
Std. dev. of X	13.7	13.5	13.7
n(Transport facilities (area) within 15min > 0)	571	554	516
n(Clusters)	28	28	27
N	714	694	645

Note: Transport facilities area is the area (in 1,000 square meters) within 15 minutes of residence that is allocated to transport facilities land use. Models and adjustments are otherwise the same as in Equation (1) and Table A4. Standard errors in (2)–(6) clustered at subzones. Significance levels: ^{c} 0.1 ^{b} 0.05 ^{a} 0.01.

Table D14. PFBS and transport facilities within 20 min of residence

	Dependent variable is PFBS (ng/mL)		
	(1)	(2)	(3)
Transport facilities (area) within 20min	−0.005	−0.039	−0.027
	(0.028)	(0.049)	(0.046)
	[−0.063–0.052]	[−0.138–0.061]	[−0.122–0.069]
	$< p = 0.853 >$	$< p = 0.434 >$	$< p = 0.569 >$
Constant	13.502	25.879	20.916
	(18.108)	(23.328)	(28.088)
	[−23.652–50.656]	[−21.986–73.745]	[−36.819–78.651]
	$< p = 0.462 >$	$< p = 0.277 >$	$< p = 0.463 >$
R^2	0.0882	0.191	0.216
Maternal baselines	Yes	Yes	Yes
Income			Yes
Area fixed effects: Planning area	Yes		
Area fixed effects: Subzone		Yes	Yes
Mean Dep Var.	24.1	23.9	23.7
Std. dev. of X	31.2	31.0	31.1
n(Transport facilities (area) within 20min > 0)	679	660	614
n(Clusters)	28	28	27
N	714	694	645

Note: Transport facilities area is the area (in 1,000 square meters) witin 20 minutes of residence that is allocated to transport facilities land use. Models and adjustments are otherwise the same as in Equation (1) and Table A4. Standard errors in (2)–(6) clustered at subzones. Significance levels: ^{c} 0.1 ^{b} 0.05 ^{a} 0.01.

E. Movers and future exposure

This appendix details how we focus on the subset of known movers in the GUSTO cohort since the start of the study and assess whether their future exposure through future residence is able to predict their past plasma PFBS concentration.

The fundamental concern is that the choice of residence is rarely random. Some unmeasured family characteristics might potentially correlate with lifestyle habits and non-point PFAS exposures which at the same time determines residence in a way that systematically leads to residential proximity to transport facilities, such locating near edges of neighborhoods or arterial roads. To help rule out this channel of confounding, we exploit the trail of GUSTO residence into the future (around 2022). Figure E28 provides a graph of this approach where there should be no association between future exposure and the (past) PFAS measures.[102]

We identify movers based on changes in reported residential addresses. More than half of the GUSTO participants in our sample (59%) have moved since the start of the study around 2009. We use the latest known residential address of these "movers" to compute the exposure to transport facilities, which is as defined in Section 2.3, except that residence is based on the latest residence and the spatial distribution of transport facilities is based on the 2019 versions. The plasma PFBS measurement remains the same version as the one collected during the start of the study around 2009. The regions, planning areas, and subzones are also based on the 2019 versions by mapping the postal codes onto the 2019 vector data.

Table E15 starts by repeating the model estimations defined in Equation (1) and reported in Table A4, but only for participants who moved in the future. We see that the associations are broadly comparable to the full sample (Table A4). For Model 2, the estimated association is 0.174 (SE 0.073, p = .025, Table E15) which is similar to the estimated association from the main analyses (0.153, SE 0.056, p = .019, Table A4). The estimated association from Model 3 is similar (0.174, SE 0.098, p = .090, Table E15).

Table E16 then reports the estimated associations between future exposure, as a negative control exposure, where we know that future exposure should not be able to predict pass plasma PFBS concentration through some unmeasured family characteristic(s) determining location choice. We do not see any statistically significant association for any model. The estimated coefficient under Model 2 is negative but not statistically significant (-0.057, SE 0.115, p = .623, Table E16). Model 3 has similar null findings (-0.075, SE = 0.132, p = .576, Table E16).

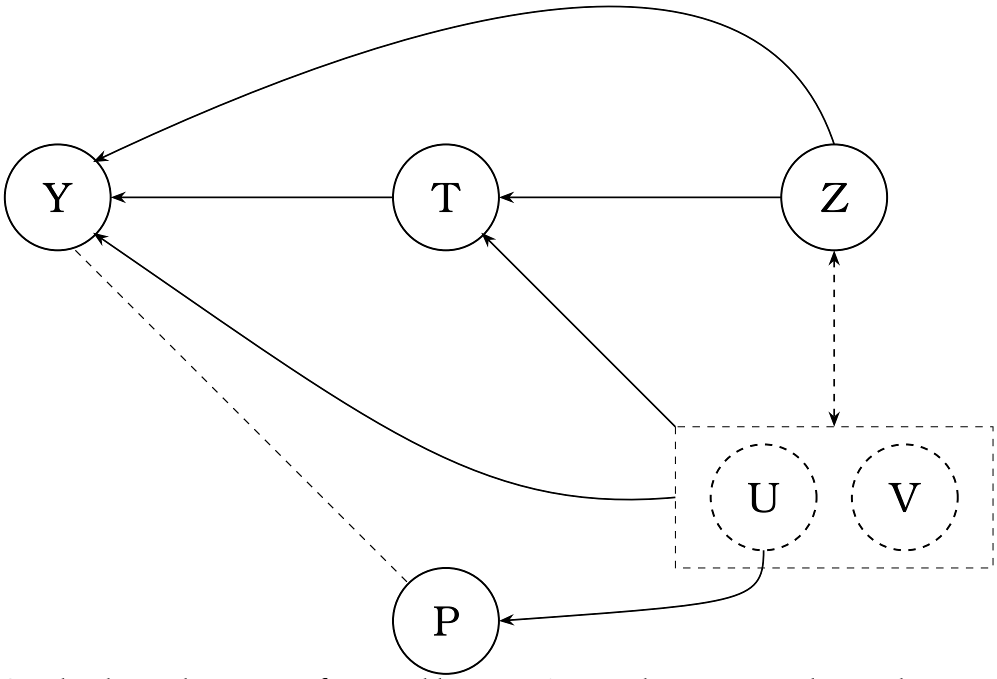

Figure E28. Placebo evaluation via future addresses. Our study is interested in evaluating the relation between plasma PFAS measures (Y) and exposure to transport facilities (T). Our models account for certain (observable) baselines (Z; Section 2.5). Our models omit certain unobserved characteristics (U and V) linked to both Y and T (and potentially Z). Part of this unmeasured baseline may capture families residential choice that might affect T (U). This U should also affect future exposure to transport facilities (P) but otherwise have no relationship to past plasma PFAS concentration (Y). V is an unobserved confounder that is intractable.

Table E15. PFBS and area of transport facilities within 500m radius (Movers only).

	Dep. var. is PFBS		
	(1)	(2)	(3)
Transport facilities (area) within 500m buffer	0.197^a	0.173^b	0.174^c
	(0.050)	(0.074)	(0.096)
	[0.095–0.299]	[0.021–0.325]	[−0.024–0.373]
	$< p = 0.000 >$	$< p = 0.027 >$	$< p = 0.082 >$
Constant	−1.712	3.989	11.303
	(21.190)	(26.236)	(34.999)
	[−45.190–41.765]	[−49.939–57.917]	[−60.930–83.536]
	$< p = 0.936 >$	$< p = 0.880 >$	$< p = 0.750 >$
R^2	0.135	0.289	0.319
Maternal baselines	Yes	Yes	Yes
Income			Yes
Area fixed effects: Planning area	Yes		
Area fixed effects: Subzone		Yes	Yes
Mean Dep Var.	24.1	24.1	23.6
Std. dev. of X	9.9	9.9	10.0
n(Transport facilities (area) within 500m buffer > 0)	258	238	219
n(Clusters)	28	27	25
N	435	401	367

Note: Sample includes only known movers in the GUSTO cohort. Table is otherwise identical to Table A4. Dependent variable is PFBS (Perfluorobutanesulfonic acid) of mothers in the GUSTO sample. Transport facilities area within 500m buffer is the area (in 1,000 square meters) of the buffer area residence that is allocated to transport facilities land use. Maternal characteristics include (i) age at delivery (quadratic), (ii) ethnicity, (iii) education, (iv) occupation, (v) marital status, and (vi) housing type. Income includes mother's income and household income (binned). Standard errors in (2)–(6) clustered at subzones. Significance levels: ^{c} 0.1 ^{b} 0.05 ^{a} 0.01.

Table E16. PFBS and area of transport facilities within 500m radius (using future exposure).

	Dep. var. is PFBS		
	(1)	(2)	(3)
Transport facilities (area) within 500m buffer	−0.108	−0.058	−0.078
	(0.078)	(0.113)	(0.130)
	[−0.269–0.052]	[−0.292–0.175]	[−0.346–0.190]
	$< p = 0.176 >$	$< p = 0.611 >$	$< p = 0.553 >$
Constant	13.003	−14.546	−33.956
	(23.237)	(28.154)	(36.456)
	[−34.854–60.860]	[−72.652–43.560]	[−109.196–41.285]
	$< p = 0.581 >$	$< p = 0.610 >$	$< p = 0.361 >$
R^2	0.0928	0.285	0.342
Maternal characteristics	Yes	Yes	Yes
Income			Yes
Planning area fixed effects	Yes		
Subzone fixed effects		Yes	Yes
Mean of dep. var.	24.1	24.0	23.8
Std. dev. of X	6.5	6.5	6.6
Clusters	26	25	25
N	443	395	364

Note: Sample includes only known movers in the GUSTO cohort. The transport facilities area is based on the future addresses of GUSTO participants and not those around time of delivery. Table is otherwise identical to Table A4. See Table E15 for the same table but for movers only using addresses at time of delivery. Dependent variable is PFBS (Perfluorobutanesulfonic acid) of mothers in the GUSTO sample. Transport facilities area within 500m buffer is the area (in 1,000 square meters) of the buffer area residence that is allocated to transport facilities land use. Maternal characteristics include (i) age at delivery (quadratic), (ii) ethnicity, (iii) education, (iv) occupation, (v) marital status, and (vi) housing type. Income includes mother's income and household income (binned). Standard errors in (2)–(6) clustered at subzones. Significance levels: ^{c} 0.1 ^{b} 0.05 ^{a} 0.01.

F. Other PFAS

In this appendix, we test and report associations between exposure to transport facilities and the other well-measured PFAS concentrations.

F.A GUSTO PFAS measurements

The different PFAS substances have different measurement ranges (Table A2). For ease of reporting, we scale the PFAS measurements by dividing by the standard deviation

$$\text{substance}^{(\text{scaled})} = \frac{\text{substance}}{\sigma_{(\text{substance})}} \tag{F4}$$

so that the interpretation of regression coefficients is how much of a standard deviation change in an input is associated with some standard deviation change in the output. For the estimates in Figure 4, where we estimate the association of exposure to transport facilities and the other PFAS substances, we also scale the area of transport facilities within residence for ease of reporting in the same scale. Importantly, we note that this linear scaling of the measurements does not aid causal inference, and separately, does not affect the t-statistics or p-values.

Figure F29 reports the correlation between perfluorobutane sulfonic acid (PFBS) and the other plasma PFAS concentrations. All measurements are scaled as described above, and the correlation is conditional on area (subzones) of the GUSTO participant. Measurements below the limit of detection (LOD) and the limit of quantification (LOQ) are first imputed by the LOD/LOQ values divided by $\sqrt{2}$. Of the other seven PFAS measurements with detection rates for at least 95% of participants (Table A2), we note a positive correlation between the plasma PFBS concentration and perfluorohexane sulfonic acid (PFHxS) perfluoroundecanoic acid (PFUnDA), and perfluorodecanoic acid (PFDA).

To test the association between the other seven plasma PFAS concentrations and exposure to transport facilities around area, we estimate

$$\begin{aligned}\text{substance}_{ic}^{(\text{scaled})} = \beta(&\text{Exposure to transport facilities})_{ic}^{(\text{scaled})} \\ &+ \gamma X_i + \delta_c(\text{subzone})_c + \varepsilon_{ic},\end{aligned} \tag{F5}$$

where $\text{substance}_{ic}^{(\text{scaled})}$ is the PFAS substance (indicated in the first column). The specification is otherwise identical to that in Equation (1) (to model (5) of Table A4). The PFAS substances and the exposure to transport facilities variable are scaled to have a standard deviation of one so that $\hat{\beta}$ is interpreted as how a standard deviation change in exposure to transport facilities is associated with a $\hat{\beta}$-times standard deviation change in the substance. Figure 4 reports the results.

Figure F30 reports the dose-response results from Equation (2) for all of the eight

PFAS measures with scaled units. We see that higher thresholds are linked to higher associations for PFBS in Figure 5 (and in Figure F30 with scaled units). We observe similar patterns with PFNA, PFOS, and PFDA.

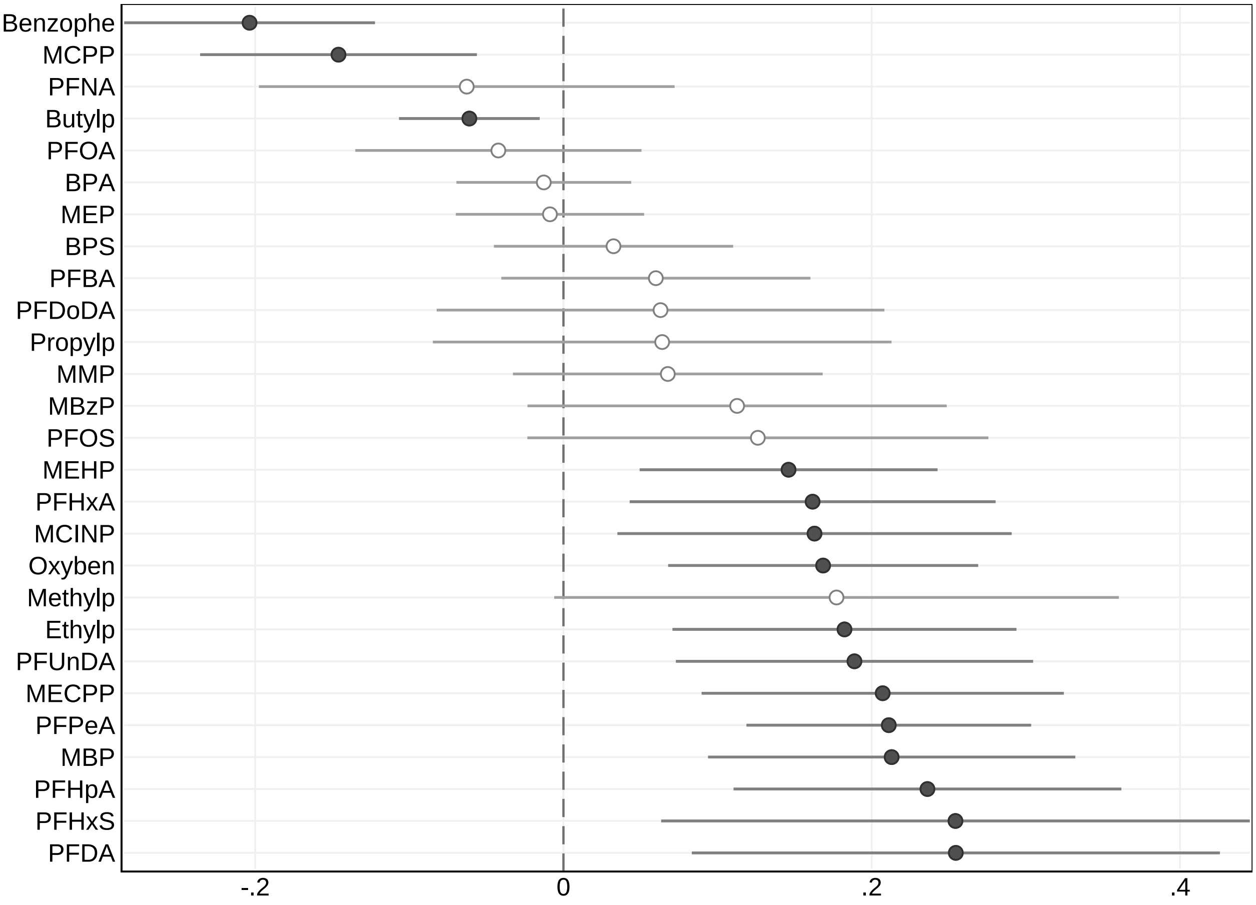

Figure F29. Correlation of PFBS (measured from cord blood) with other PFAS substances. Horizontal axis reports the $\hat{\beta}$ coefficients from estimating

$$\text{substance}_{ic}^{\text{(scaled)}} = \beta \text{PFBS}_{ic}^{\text{(scaled)}} + \delta_c(\text{subzone})_c + \varepsilon_{ic},$$

where substance$_{ic}$ is one of the PFAS substances indicated on the vertical axis. Estimates significant at the 5% level have black markers; the remaining estimates have hollow markers. Gray horizontal lines are the 95% confidence intervals constructed from standard errors clustered at planning areas. Measurements that are below LOD and LOQ values are first imputed (Table A2) and then scaled to have a standard deviation of one.

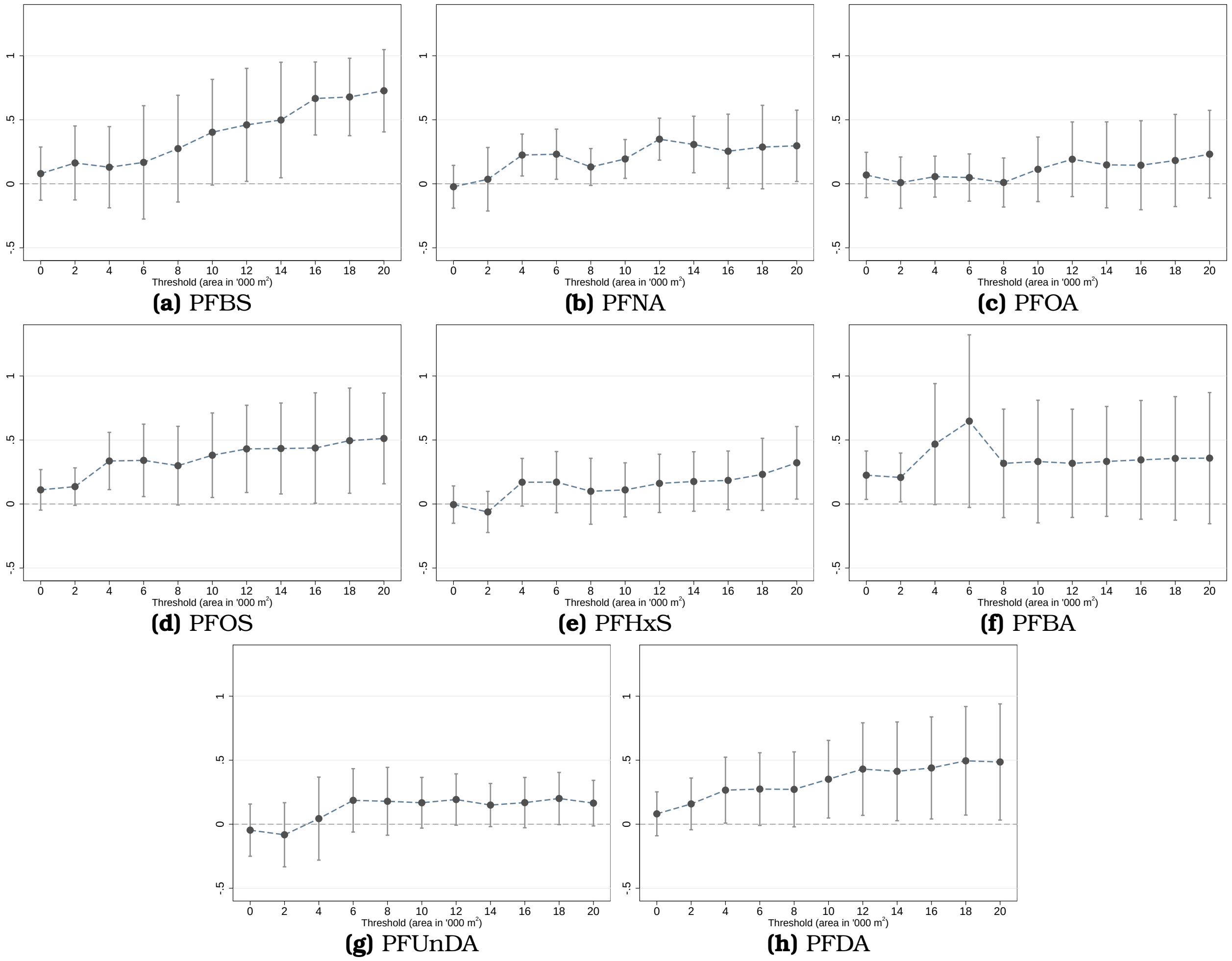

(a) PFBS **(b)** PFNA **(c)** PFOA **(d)** PFOS **(e)** PFHxS **(f)** PFBA **(g)** PFUnDA **(h)** PFDA

Figure F30. Thresholds for other PFAS. PFAS measurements and the exposure measure have been scaled to have unit standard deviation. Area of transport facilities based on 500m radius.

F.B S-PRESTO PFAS measurements

We use participants from S-PRESTO, a separate cohort study in Singapore, as supplementary data for additional cross-sectional analyses. The key relevant detail to the present study is that S-PRESTO recruited participants between 2015–2017, and therefore offers a different time point for evaluation.

The S-PRESTO (Singapore Preconception Study of Long-Term Maternal and Child Outcomes) recruited N = 1,039 ethnically-diverse (Chinese, Malay, or Indian) participants aged 18–45 who intended to get pregnant and deliver in Singapore between February 2015–October 2017. Similar to GUSTO, S-PRESTO is inclusive but there were exclusion criteria for to-be mothers with existing treatments and conditions (e.g., undergoing fertility treatment or had diabetes) so that the sample included mostly healthy mother-child pairs.[3,70]

Plasma PFAS concentrations from S-PRESTO come from a preconception plasma blood sample collected at enrollment around 2015–2017. S-PRESTO collected only maternal blood so we lack the same PFAS measurements from cord blood as a reflection of neonatal exposure. The samples were sent for testing around 2021 for 15 PFAS: perfluorohexanesulfonic acid (PFHxS), linear and branched perfluorooctanesulfonic acid (PFOS), linear perfluorooctanoic acid (PFOA), perfluorononanoic acid (PFNA), perfluoroheptanesulfonic acid (PFHpS), perfluorodecanoic acid (PFDA), perfluoroheptanoic acid (PFHpA), N-methylperfluorooctanesulfonamido acetic acid (NMeFOSAA), 6:2 polyfluoroalkyl phosphate ester (6:2 PAP), 6:2 fluorotelomer phosphate diester (6:2 diPAP), perfluorobutanesulfonic acid (PFBS), 6:2 fluorotelomer sulfonate (6:2 FTS), N-ethyl perfluorooctanesulfonamidoacetic acid (NEtFOSAA), and perfluorodecanesulfonic acid (PFDS), and perfluorooctanesulfonamide (PFOSA). The measurement data came with the following LOD values replaced with half of the LOD value, and this is what we use. Table F17 reports the completeness of PFAS detection and the corresponding LOD values ranked by completeness. For this set of analyses, we include the first eight measurements (seven PFAS) with detection in at least 34% of the participants. The remaining PFAS are excluded. The seven included PFAS (LOD) are: PFHxS (0.10 ng/ml), PFOS (0.20 ng/ml), PFOA (0.50 ng/ml), PFNA (0.50 ng/ml), PFHpS (0.20 ng/ml), PFDA (0.50 ng/ml), and PFHpA (0.20 ng/ml).

As far as possible, we adjust for maternal baselines in the S-PRESTO evaluations so that they are comparable to our evaluations using the GUSTO sample. For the S-PRESTO evaluations, we adjusts for age of mother during recruitment (flexibly in quadratics), education, employment, marital status, ethnicity, and household income. We note that these adjustments do not map exactly to those in the GUSTO sample. Household income, for instance, would have different stratas compared to those asked in the earlier GUSTO recruitment. All other specifications and adjustments for unmeasured spatial heterogeneity are otherwise similar as defined in Equation (1). We are unable to focus on

Table F17. Completeness of S-PRESTO plasma PFAS measurements

Full analyte name	Shorthand	Measured	< LOD	LOD value
Perfluorohexane-1-sulphonic acid	PFHxS	384	0	0.1 ng/ml
Perfluorooctanesulfonic acid	PFOS-Linear	384	0	0.2 ng/ml
Perfluorooctanesulfonic acid	PFOS-Branched	383	1	0.2 ng/ml
Perfluorooctanoic acid	PFOA-Linear	374	10	0.5 ng/ml
Perfluorononanoic acid	PFNA	233	151	0.5 ng/ml
Perfluoroheptanesulfonic acid	PFHpS	186	198	0.2 ng/ml
Perfluorodecanoic acid	PFDA	159	225	0.5 ng/ml
Perluoroheptanoic acid	PFHpA	130	254	0.2 ng/ml
2-(N-Methylperfluorooctanesulfonamido) acetic acid	NMeFOSAA	6	378	0.1 ng/ml
6:2 Fluorotelomer phosphate monoester	6:2 PAP	5	379	0.1 ng/ml
6:2 Fluorotelomer phosphate diester	6:2 diPAP	2	382	0.1 ng/ml
Perfluorobutanesulfonic acid	PFBS	1	383	0.2 ng/ml
6:2 Fluorotelomer sulfonic acid	6:2 FTS	0	384	0.1 ng/ml
2-(N-Ethylperfluorooctanesulfonamido) acetic acid	NEtFOSAA	0	384	0.2 ng/ml
Perfluorodecanesulfonic acid	PFDS	0	384	0.1 ng/ml
Perfluorooctanesulfonamide	PFOSA	0	384	0.1 ng/ml

Note: Table reports the completeness of plasma PFAS measurements in the S-PRESTO sample (n = 384). Measurements in nanograms per millilitre (ng/ML). LOD = limit of detection.

the PFBS (perfluorobutanesulfonic acid) since only one sample had successful detection above the LOD (Table F17). These estimates are reported in Figure F31.[103]

The exposure to transport facilities measure is as defined in Section 2.3, except that we map the residence of the S-PRESTO participants to the 2014 land use plans. Likewise, the regions, planning areas, and subzones are also based on the 2014 versions by mapping the postal codes onto the 2014 vector data.

PFAS	Full analyte name	N	< LOD	LOD value	Est. (95% Conf. Int.)	P-value
PFHxS	Perfluorohexane-1-sulphonic acid	384	0	0.1 ng/ml	0.038(-0.021 to 0.097)	0.202
PFOS-Linear	Perfluorooctanesulfonic acid	384	0	0.2 ng/ml	0.100(0.026 to 0.175)	0.01[b]
PFOS-Branched	Perfluorooctanesulfonic acid	383	1	0.2 ng/ml	0.061(-0.038 to 0.160)	0.217
PFOA-Linear	Perfluorooctanoic acid	374	10	0.5 ng/ml	0.028(-0.050 to 0.106)	0.469
PFNA	Perfluorononanoic acid	233	151	0.5 ng/ml	0.112(0.013 to 0.212)	0.028[b]
PFHpS	Perfluoroheptanesulfonic acid	186	198	0.2 ng/ml	0.097(0.014 to 0.180)	0.024[b]
PFDA	Perfluorodecanoic acid	159	225	0.5 ng/ml	0.031(-0.014 to 0.076)	0.166
PFHpA	Perluoroheptanoic acid	130	254	0.2 ng/ml	0.124(0.044 to 0.204)	0.004[a]

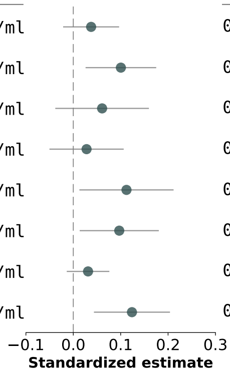

Figure F31. Figure reports estimates for PFAS measurements from the S-PRESTO cohort study. Horizontal axis of the plot reports the $\hat{\beta}$ coefficients from estimating

$$\text{PFAS}ic^{\text{(scaled)}} = \beta(\text{Exposure to transport facilities})ic^{\text{(scaled)}} + \gamma X_i + \delta_c(\text{subzone})c + \varepsilon ic,$$

where $\text{PFAS}_{ic}^{\text{(scaled)}}$ is the PFAS substance (indicated in the first column). Transport facilities and regional tags are based on the 2014 versions. All models adjust for mother's age (in quadratics), employment status, education, marital status, ethnicity, and household income collected from the S-PRESTO sample. Gray horizontal lines are the 95% confidence intervals constructed from standard errors clustered at planning areas. Significance levels: ^{c} 0.1 ^{b} 0.05 ^{a} 0.01.
